# Effects of fluoride and lead on enamel composition during the maturation stage of amelogenesis in rat mandibular third molars

**DOI:** 10.1007/s00223-026-01511-z

**Published:** 2026-04-02

**Authors:** J. Tostes-Figueiredo, N. Macedo-Ribeiro, G. H. L. Santos, I. M. Porto, R. F. Gerlach, F. B. de Sousa

**Affiliations:** 1 Department of Basic and Oral Biology, Faculty of Dentistry of Ribeirao Preto, University of Sao Paulo (DBBO/FORP/USP), Ribeirao Preto, Sao Paulo Brazil; 2 Department of Morphology and Children’s Clinic, Sao Paulo State University (FOAr-UNESP), Araraquara, Sao Paulo Brazil; 3 Department of Morphology, Health Sciences Center, Federal University of Paraiba (UFPB), Joao Pessoa, Paraiba Brazil

**Keywords:** Hypomineralization, Dental enamel, Environmental contaminants, Fluorosis, Amelogenesis

## Abstract

Dental enamel formation depends on tightly regulated mineral deposition and organic matrix removal during the maturation stage. Fluoride (F) adversely affects the maturation stage of amelogenesis, causing hypomineralized enamel defects. However, hypomineralized enamel defects have increased worldwide in recent years, making it urgent to understand how co-exposure to other environmental contaminants affects fluorosis and maturation-stage amelogenesis at fluoride levels sufficient to induce defects during early-life exposure conditions in experimental models. This study aimed to determine whether lead (Pb) co-exposure amplifies fluoride-induced disturbances in the enamel maturation stage by mapping depth-dependent changes in mineral, organic, and water components across the enamel layer. Pregnant Wistar rats and their offspring were exposed to F (50 ppm), Pb (30 ppm), or both in drinking water from gestation to postnatal day 30. Mandibular third molars were evaluated using a modified Thylstrup–Fejerskov index and analyzed by quantitative microradiography and polarized light microscopy to obtain depth-resolved compositional data. Mean defect scores were 0.0 (Control), 2.6 (F), and 3.2 (Pb + F). Entire enamel thickness mineral volume decreased from 71.0% (Control) to 41.1% (F) and to 35.2% (Pb + F), accompanied by a marked increase in organic matrix volume (9.3% to > 30%) (*p* < 0.0001). Spatial analyses showed minimal disturbances and smaller effect sizes at the superficial region, with progressively larger effects toward the central and inner enamel regions. These findings demonstrate that Pb co-exposure exacerbates fluoride-induced enamel hypomineralization and disrupts the normal spatial pattern of enamel maturation, emphasizing the importance of combined environmental exposures for understanding mechanisms governing the development and maturation of mineralized tissues.

**Supplementary Information:**

The online version contains supplementary material available at 10.1007/s00223-026-01511-z.

## Introduction

Enamel formation is a highly regulated biological process that involves sequential stages of matrix secretion, mineral deposition, and maturation, culminating in the replacement of organic material by densely packed apatite crystals [1]. Disruption at any of these stages may impair amelogenesis and result in developmental enamel defects, particularly hypomineralization [2]. Both genetic and environmental factors influence enamel development and determine susceptibility to such defects [3].

Among environmental agents, fluoride plays a dual role. At optimal levels, it is effective in caries prevention; however, excessive fluoride intake during tooth development leads to dental fluorosis, a hypomineralizing condition that arises pre-eruptively, predominantly during the maturation stage of amelogenesis [4]. Fluorotic enamel is characterized by subsurface porosity, increased water content, and retention of organic matrix proteins, despite apparently preserved secretion of enamel proteases [5]. Rather than directly inhibiting enamel matrix proteinases, fluoride is thought to delay matrix removal indirectly by altering the mineralizing environment, including calcium availability and pH regulation [4, 6]. In addition, fluoride disrupts ameloblast intracellular Ca^2+^ homeostasis and induces endoplasmic reticulum stress, impairing cellular functions essential for enamel maturation [7].

Although fluoride is the environmental contaminant mostconsistently associated with enamel hypomineralization, other toxicants may also interfere with amelogenesis. Lead (Pb) is a non-essential heavy metal that accumulates in mineralized tissues due to its chemical similarity to calcium, thereby disturbing metal homeostasis [8, 9]. During tooth development, Pb can be incorporated into enamel and dentin [10, 11] and has been shown to inhibit enamel matrix proteolysis in vitro, particularly affecting zinc-dependent enzymes such as MMP-20 [12].

Increasing evidence suggests that combined exposure to fluoride and Pb may amplify their individual toxic effects. Experimental studies have shown that fluoride increases Pb bioavailability and accumulation in blood and calcified tissues [13], raising the possibility that co-exposure may exacerbate disturbances during enamel maturation. Indeed, previous work demonstrated that Pb aggravates fluoride-induced fluorosis severity in rat incisors [14]. However, these studies relied primarily on assessments of the enamel surface and did not address how co-exposure affects the spatial distribution of mineral and organic components across the enamel thickness.

Enamel mineralization depends on the coordinated degradation and removal of the organic matrix by two major proteases: MMP-20 during the secretory stage and KLK4 during maturation [15]. Disruption of this functional overlap results in protein retention and region-specific hypomineralization, as demonstrated in genetic models of protease deficiency. Because enamel maturation proceeds heterogeneously across the enamel thickness, spatially resolved compositional analyses are essential to understand how environmental toxicants interfere with this process.

Rodent incisors are commonly used to study enamel hypomineralization; however, their complex prism decussation pattern and the presence of a superficial iron-rich layer limit accurate quantification of mineral, organic, and water volumes [16, 17]. In contrast, rat molars lack this iron-rich surface layer and exhibit a more favorable prism orientation, allowing reliable depth-resolved compositional analyses. Notably, mandibular third molars in young rats complete enamel maturation after weaning; however, exposure to contaminants such as lead and fluoride may already occur prenatally and during lactation, with differing transfer dynamics. During lactation, the pups are exposed to lower levels of fluoride [18].

Therefore, the present study aimed to determine whether Pb co-exposure exacerbates fluoride-induced enamel hypomineralization and to quantify, with spatial resolution, the mineral, organic, and water volumes across the enamel thickness. Using mandibular third molars from 30-day-old rats exposed to fluoride, lead, or both from gestation onward, we sought to characterize region-specific compositional signatures of disturbed amelogenesis and to evaluate the relevance of combined environmental exposures in the pathogenesis of developmental enamel defects.

## Materials and methods

### Ethical approval and animal care

All experimental procedures complied with the ethical guidelines of the Institutional Ethics Committee for the Use of Animals in Research (CEUA–FORP/USP, protocol no. 2022.1.7.58.0) and followed the Guide for the Care and Use of Laboratory Animals issued by the National Council for the Control of Animal Experimentation.

Twelve Wistar rats (8 females and 4 males; 7 weeks old; 150–170 g) were obtained from the institutional breeding facility and, after a brief acclimatization period, randomly assigned at the onset of gestation to four experimental groups (two females and one male per group): Control (filtered water), F (50 ppm fluoride as fluosilicic acid, H_2_SiF_6_), Pb (30 ppm lead as lead acetate, Pb(CH_3_COO)_2_·3 H_2_O), and Pb + F (50 ppm fluoride plus 30 ppm lead).

The animals were housed under controlled conditions (12-h light/dark cycle, 25 °C) with ad libitum access to standard chow and water. Weekly body weight (Fig. S1) and daily water intake (Fig. S2) were monitored.

The offspring were born 3–5 weeks after exposure onset and, after weaning, continued receiving the same treatments as their dams. At 30 days of age, rats were euthanized under deep anesthesia, mandibles were collected and frozen for analysis, and bone samples were obtained for Pb and fluoride quantification, with results presented in the supplementary material (Figs. S3–S4).

### Tooth examination and fluorosis score assessment

After euthanasia, molars were carefully extracted from the mandibles, cleaned, air-dried, and visually inspected under a stereomicroscope. Enamel opacities and surface defects were identified, particularly on the mesial surface of mandibular third molars in the most affected groups. The mesial surfaces were photographed at FORP-USP using a Canon EOS Rebel T6i equipped with a 100 mm macro lens and extension tube, and ten lower third molars per group were analyzed.

To objectively assess the severity of fluoride-induced enamel alterations, a fluorosis scoring system was applied. Because the macroscopic features resembled human dental fluorosis described by Thylstrup and Fejerskov [19], a lesion score modified from the Thylstrup–Fejerskov (TF) index [19] was developed for rat third molars, considering the specific characteristics observed in this model (Table 1).

**Table 1:** Fluorosis score (Modified Thylstrup–Fejerskov (TF) index)

Score	Description
0	Normal enamel without alterations
1	Opacity affecting less than 50% of the surface
2	Opacity affecting more than 50% of the surface
3	Opacity and focal enamel loss
4	Opacity and enamel loss in bands
5	Opacity and enamel loss affecting more than 50% of the surface

**Fig. 1:**
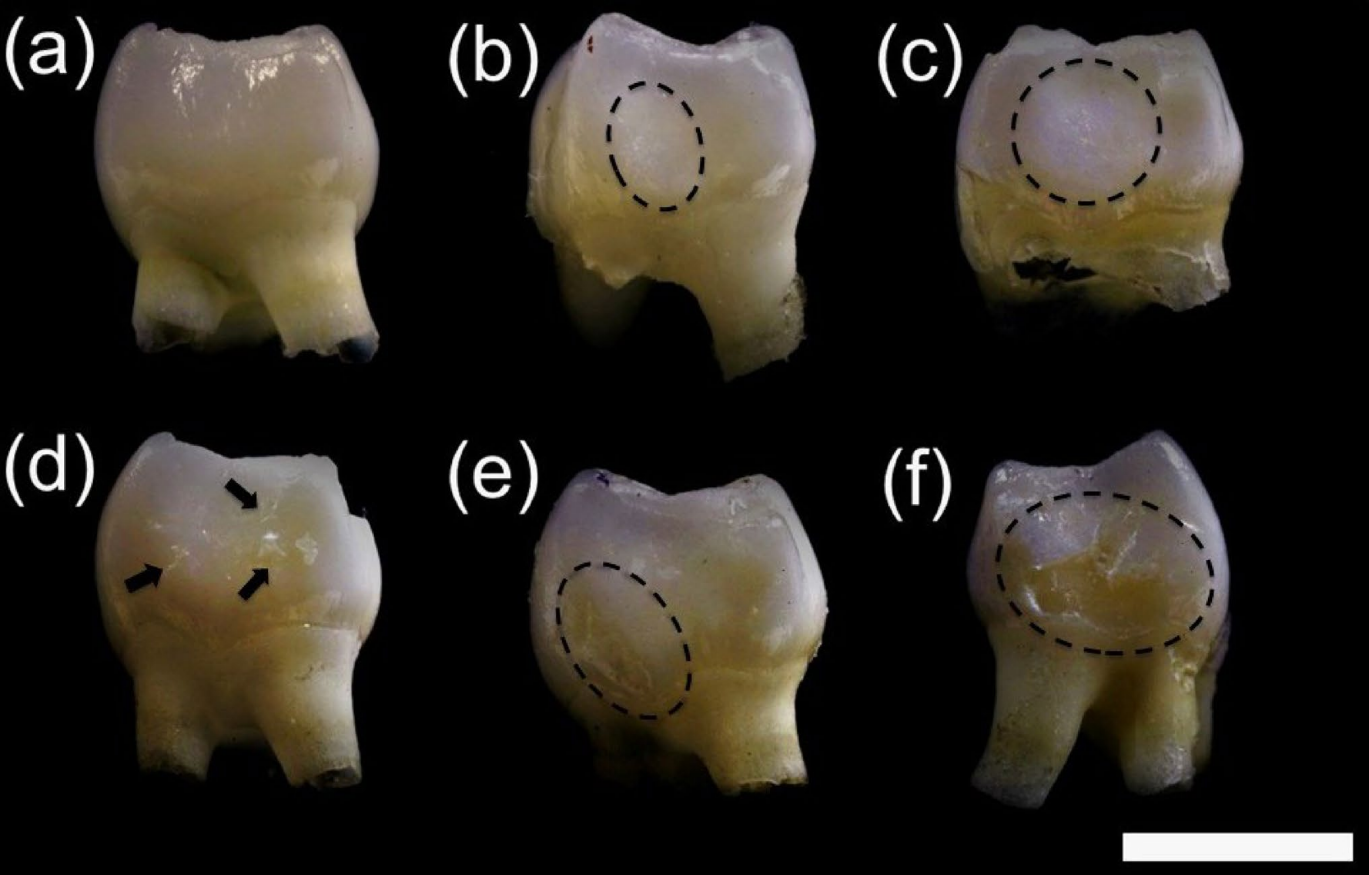
Mesial surfaces of 30-day-old mandibular 3rd molar teeth illustrating the enamel defect scores used in this study. **a** Score 0: normal enamel without alterations; **b** score 1: opacity affecting less than 50% of the surface (highlighted by a black dotted circle); **c** score 2: opacity affecting more than 50% of the surface (highlighted by a black dotted circle); **d** score 3: opacity associated with focal enamel loss (indicated by a black arrow); **e** score 4: opacity with enamel loss in horizontal bands (highlighted by a black dotted circle); and **f** score 5: opacity with enamel loss affecting more than 50% of the surface (highlighted by a black dotted circle). Bar = 1 mm

This scoring system categorized teeth according to fluorosis severity, ranging from mild opacities to marked enamel loss. Representative examples of each score are shown in Fig. 1, illustrating the gradient of opacity and structural disruption across experimental groups. All teeth were evaluated independently by two blinded examiners.

Dental enamel microstructure and elemental composition were analyzed by SEM–EDS (JSM-6610LV, JEOL, Tokyo, Japan) under low-vacuumconditions without coating. Elemental data were acquired using AZtec software (Oxford Instruments, Oxford, UK). Detailed procedures are described in the Supplementary Material. Surface elemental mapping of incisors and molars was performed to determine the presence of iron, and line scans across molar cross-sections were conducted to evaluate calcium and carbon distribution throughout the enamel thickness.

### Preparation of ground sections

Undemineralized, unfixed longitudinal ground Sects. (80–100 μm thick) were prepared from each tooth following established protocols [20]. Dental slices (~ 300 μm thick) were sectioned under continuous water irrigation using a diamond disc and thinned to the final thickness using a precision grinding device and silicon carbide papers.

Final section thickness was verified at the histological sites of interest by positioning the specimens edge-on under a polarizing microscope equipped with a 20× objective and an eyepiece reticle (0.7 μm resolution). All sections were stored in 0.02% aqueous sodium azide (NaN_3_) until analysis.

### Mineral volume quantification

Quantitative microradiographic analysis was performed using a digital X-ray camera coupled to a high-resolution micro-computed tomography system (Skyscan 1172, Bruker, Belgium), operated at 60 kV (peak energy 10 keV), with flat-field correction, no additional filters, and a pixel size of 0.94 μm.

Each section was scanned together with an aluminum step-wedge consisting of ten high-purity foils (99.9%; ESPI Chemicals, USA), each 20 μm thick, providing a calibration range of 20–200 μm. Based on the X-ray energy, aluminum density (2.7 g/cm^3^), and the empirical formula and density of enamel mineral [21], linear attenuation coefficients were calculated for aluminum (70.740 cm^−1^) and enamel mineral (134.017 cm^−1^). Calibration curves were obtained by non-linear regression between aluminum thickness and grayscale values, and then the absolute mineral volume was measured.

**Fig. 2:**
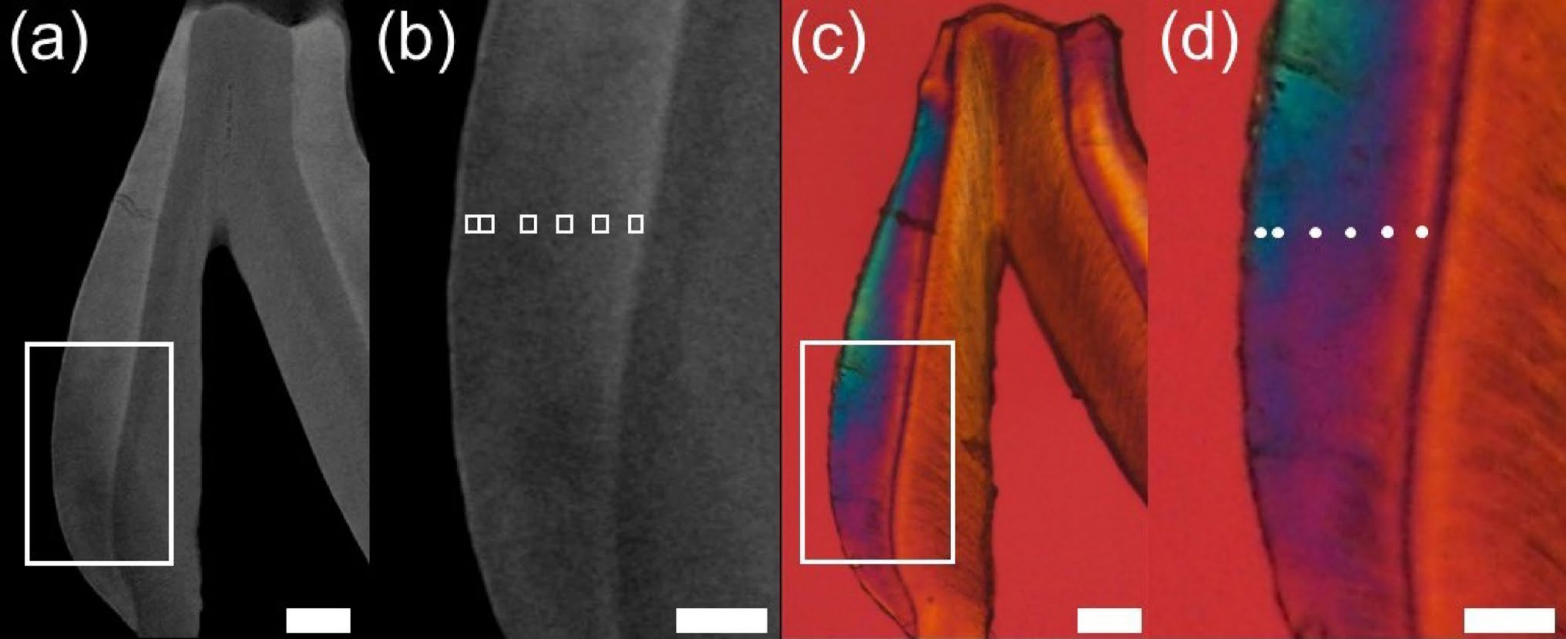
Microradiographs (**a**–**b**) and birefringence image of polarized light (**c**–**d**) analyses made in ground sections of lower 3rd molars of 30-day-old animals. **a** Microradiograph picture showing a rectangle amplified in (**b**). **b** White points where the 6 measurements (10 × 10 μm each) were made at the following distances from the enamel surface: 7, 15, 40, 60, 80, and 100 μm. **c** Polarized light picture (crossed polarizers and a Red I filter is in place), with the rectangle showing the area of the dental enamel with positive birefringence amplified in (**d**). **d** The 6 points where the phase retardation was measured using the Berek compensator (*n* = 5 in each of the 2 wavelengths used). Based on the results obtained in microradiography and polarized light measurements, mineral, organic, and water volumes (%) were obtained. Scale bar: (**a**) and (**c**): 100 μm; (**b**) and (**d**): 50 μm–

To capture spatial variation across the enamel thickness, six standardized histological sites were selected along a line parallel to the enamel prisms. Measurements were performed at 7, 15, 40, 60, 80, and 100 μm from the enamel surface, using a fixed area of 10 × 10 μm at each site (Fig. 2). The location of the line and the histological sites was achieved by correlating histological landmarks identified in both polarizing microscopy and microradiographic images. Grayscale values obtained at each histological site were converted into mineral volume percentages using the Angmar equation [22], following the approach described by Gan et al. [23].

### Organic and water volume quantification

The quantification of absolute non-mineral components (organic and water fractions) was performed at the same histological sites previously used for mineral volume assessment. Measurements were carried out under water immersion using a polarizing microscope (Axioskop 40, Carl Zeiss, Germany) equipped with a 0–5 order Berek compensator and a 550 nm interference filter (10 nm bandwidth; Edmund Optics, USA). At each site, phase retardation was measured five times and averaged by a single trained examiner.

Birefringence sign was determined using a Red I retardation filter, and birefringence values were calculated from mean phase retardation adjusted for section thickness. Combined with mineral volume data, organic and water volumes were calculated according to the optical model described by Sousa, Vianna, and Magalhães [24] and subsequently validated by Dantas et al. [20].

In addition, enamel permeability was quantified at each histological site as previously described [25], using the ratio of squared water volume to non-mineral volume. This approach enabled differentiation of mineral, organic, and water components based on enamel birefringence behavior under polarized light.

### Statistical analyses

All analyses were performed in RStudio software (version 4.5.2).

### Sample size calculation

Sample size calculation was based on a previously published effect size (Cohen’s d of 1.4) in fluoride groups in a similar study [14]. Along with a 2-tailed significance level of 5%, a power of 80%, and a sample loss estimate of 10%, the sample size per group was 10, as calculated with the function pwr.t.test (package pwr).

### Examiner reliability

Two examiners evaluated visual surface features of the samples using a scoring system. Forty samples were analyzed twice, with a time interval of 15 days. The inter- and intra-reliabilities were tested using the function cohen.kappa (package psych).

### Descriptive and inferential statistics on component enamel volumes, and definition of enamel regions

Descriptive statistics of component volumes per group as a function of the distance from the enamel surface were calculated using the function describe (psych package). Considering that the solid component volumes are the main ones in the pathogenesis of hypomineralized developmental defects of enamel, the remaining inferential analyses were focused on two continuous outcomes (mineral and organic volumes). Because the distances from the enamel surface represent ordered spatial locations within the same experimental unit rather than independent observations, they were not analyzed as an isolated factor. To capture spatial variation while avoiding pseudo-replication, mineral and organic volume profiles were separately integrated across predefined enamel regions using area under the curve (ΔZ, vol%xµm) metrics, calculated by a trapezoidal rule. Enamel regions were defined from the enamel surface as: superficial (7–15 μm), central (40–60 μm), and close to dentin (80–100 μm), as well as composite regions corresponding to the outer enamel half (7, 15, and 40 μm), inner enamel half (60, 80, and 100 μm), and the entire enamel thickness (7–100 μm).

The aim of the inferential analyses was the interaction between treatment and enamel region, which was tested to determine whether treatment effects varied across the enamel regions, while the main effect of treatment and region was not interpreted in isolation. This was done using the function lmer (package lme4), with the syntax “lmer(DZ_outcome ~ treatment * region + (1 | ID))”, where the term ”1| ID”(ID = sample single identifier) avoids that different regions from the same sample contribute to multiple comparisons. The effect of the interaction between treatment and enamel region was calculated for each outcome. Then, pairwise comparisons between treatments within each enamel region were performed using model-based t-tests derived from the mixed-effects model, without adjustment for multiple comparisons [26]. Model-based estimated marginal means were obtained using the emmeans() function (*emmeans* package).

Pairwise contrasts between treatment groups were computed using the contrast() function (*emmeans* package) with the “pairwise” method, generating differences in regional ΔZ values between treatment groups while preserving the variance–covariance structure specified in the mixed-effects model. Following the recommendation for post hoc pairwiseanalyses planned during study design [26]. Statistical inference for these contrasts was based on t statistics derived from the fitted mixed-effects models, with degrees of freedom estimated using the Satterthwaite approximation as implemented in the *lmerTest* package. Cohen’s d effect size for each pairwise contrast was calculated by dividing the model-estimated difference between treatment means by the residual standard deviation of the corresponding mixed-effects model, obtained via the sigma() function (*lme4* package). Confidence intervals for Cohen’s d were derived by scaling the confidence limits of the model-based contrasts. The one-tailed significance level of 5% was used in all analyses.

### Inferential statistics on visual aspects of dental enamel

The effect of treatment on the visual aspect of dental enamel surface (quantified by a scoring system) was tested using the functions kruskal.test (package stats; for p value) and kruskal_effsize (package rstatix; for the effect size and its 95% confidence interval). Pairwise post-host analyses were performed using the functions pairwise_wilcox_test (package rstatix) and wilcoxonR (package rcompanion), for p value, effect size, and its 95% confidence interval, respectively.

## Results

The mean positive birefringence of dental enamel in the control group was 7.8 × 10^−4^ (± 11.6 × 10^−1^). The overall composition of the entire enamel thickness, expressed as volume and weight percentages (densities obtained from de Abreu et al. [27], is summarized in Table 2. Across groups, organic content generally exceeded water content, except in the control group, in which water represented the largest non-mineral fraction.

**Table 2:** Percentage composition of entire enamel thickness by volume and by weight

	Control	Pb (30 ppm)	F (50 ppm)	Pb + F
A. Composition by volume (%)				
Mineral (%)	71.00 ± 1.84	65.81 ± 7.10^a^	41.12 ± 8.08^ab^	35.18 ± 4.42^abc^
Organic matrix (%)	9.27 ± 1.79	14.09 ± 6.64^a^	31.55 ± 6.28^ab^	35.19 ± 5.46^abc^
Water (%)	12.77 ± 0.54	13.06 ± 1.11	20.28 ± 4.54^ab^	22.69 ± 3.90^ab^
B. Composition by weight (%)				
Mineral (%)	89.18 ± 2.31	85.72 ± 9.25	65.61 ± 12.90^ab^	59.38 ± 7.46^ab^
Organic matrix (%)	5.45 ± 1.05	8.59 ± 4.05	23.57 ± 4.69^ab^	27.81 ± 3.08^abc^
Water (%)	5.36 ± 0.23	5.69 ± 0.48	10.82 ± 2.42^ab^	12.81 ± 2.20^abc^

Values are expressed as mean ± SD

Based on the densities: 2.99 g/cm^3^ (mineral), 1.40 g/cm^3^ (organic), and 1.0 g/cm^3^ (water) [27]. ^a^statistically different from the Control group; ^b^statistically different from the Pb group; ^c^statistically different from the F group (*p* < 0.05)

**Fig. 3:**
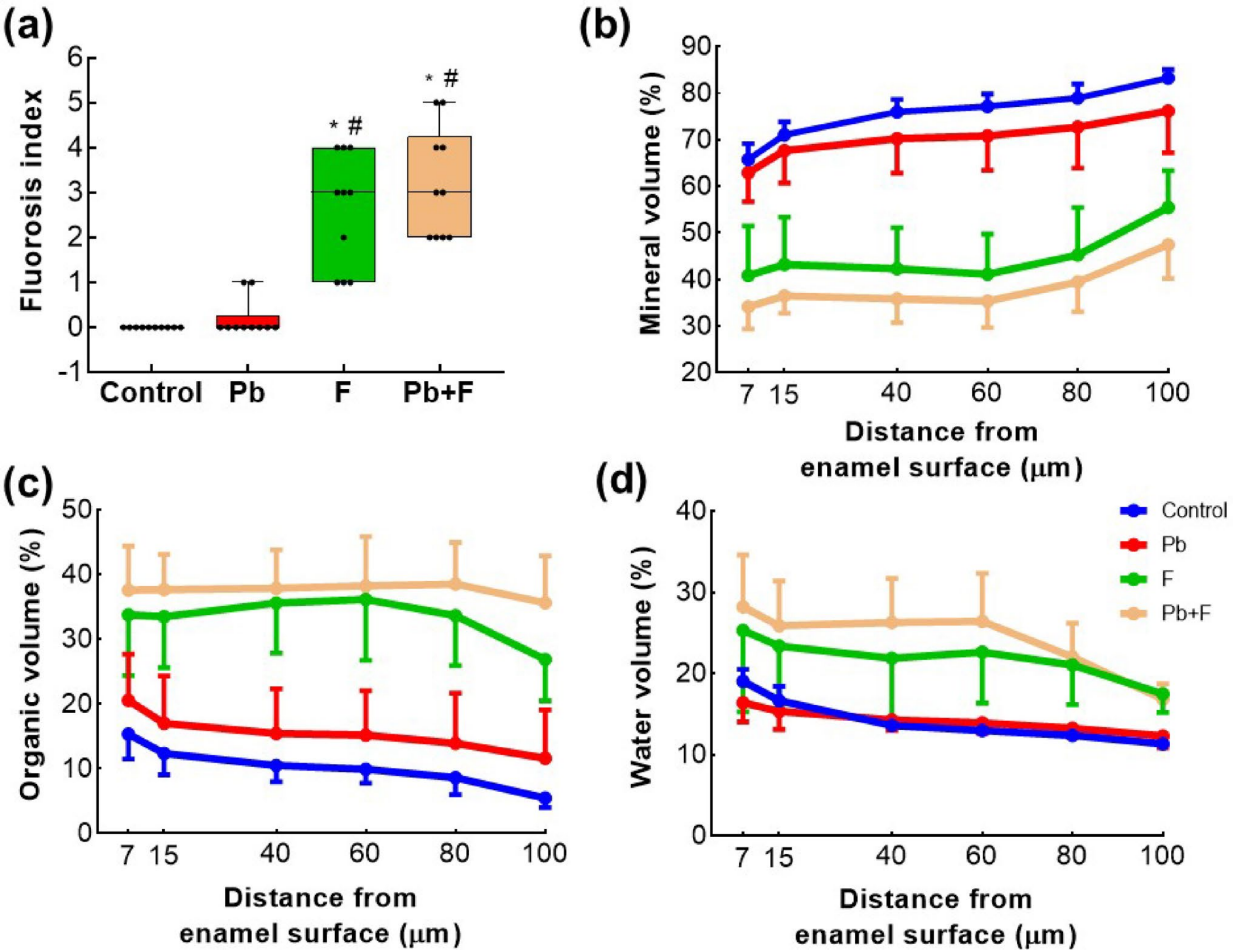
Fluorosis severity and enamel compositional changes (volume%) in mandibular third molars of 30-day-old Wistar rats. **a** Fluorosis index scores in Control, Pb, F, and Pb + F groups. Boxplots represent median and interquartile range; individual data points are shown. *p < 0.0001 vs. Control; and #*p* < 0.001 vs. Pb group. **b**–**d** Depth-dependent changes in enamel composition across the distance from the outer enamel surface to the inner enamel (7–100 μm). **b** Mineral volume (%), **c** organic matrix volume (%), and **d** water volume (%). Measurements were obtained from microradiographs and polarized light birefringence analyses performed on non-decalcified Sects.  (80–100 μm thickness) of mandibular third molars. Data are presented as mean ± SD

Depth-resolved analyses revealed distinct spatial gradients of mineral, organic, and water volumes across the enamel thickness (Fig. 3b–d). In the control group, mineral volume increased progressively from the enamel surface toward the enamel–dentin junction, accompanied by a gradual decrease in organic and water contents. This pattern was altered in fluoride-exposed groups, which exhibited a compositional slope located midway across the enamel thickness, characterized by reduced mineral content and increased organic and water volumes.

In fluoride-containing groups (F and Pb + F), mineral volume percentages were consistently lower throughout the enamel thickness, with the most pronounced reductions in the central and inner enamel regions. These reductions were accompanied by higher organic volume values across most depths, particularly in these regions. Notably, the Pb + F group showed a further increase in organic content near the enamel–dentin junction.

In contrast, the Pb-only group displayed mineral, organic, and water volume profiles largely overlapping those of the control group, with only a modest increase in organic volume in the inner enamel. Water volume showed limited depth-dependent variation among groups, although fluoride-containing groups tended to exhibit higher values near the superficial and inner enamel regions. Permeability profiles were similar across groups, showing peak values in the superficial enamel and a progressive decrease toward the inner regions (raw permeability and additional compositional data are provided in the Supplementary Material (Tables S1–S3)). Overall, these profiles indicate that fluoride exposure, alone or combined with Pb, disrupts the spatial distribution of enamel components across the full enamel thickness, whereas Pb exposure alone produces subtler alterations predominantly restricted to the inner enamel.

Regarding the quantification of visual enamel changes by the scoring system, thenon-parametric Kruskal–Wallis test revealed a significant difference among groups of third molars (H = 32.01, *p* < 0.0001), and the incisors also showed a hypomineralization phenotype (Supplementary material Fig. S5). Wilcoxon multiple comparisons showed that the fluoride (F) and co-exposure (Pb + F) groups exhibited significantly higher scores than both the control and Pb-only groups (Fig. 3a). The Pb + F group displayed the highest values, differing significantly from the Pb group (*p* = 0.000101) and from the control group (*p* < 0.0001). In contrast, no statistical difference was observed between the F and Pb + F groups. Descriptive statistics support this pattern, with mean scores of 0.0 (Control), 0.2 (Pb), 2.6 (F), and 3.2 (Pb + F), indicating a progressive increase in lesion severity according to exposure type. Examiners’ reliability was very good (intraexaminers = 0.96; interexaminers = 0.97). Representative SEM–EDS maps of third molars are presented in the Supplementary Material. Major elements were detected in both dental types, while iron was observed only in incisors (Fig. S6).

**Fig. 4:**
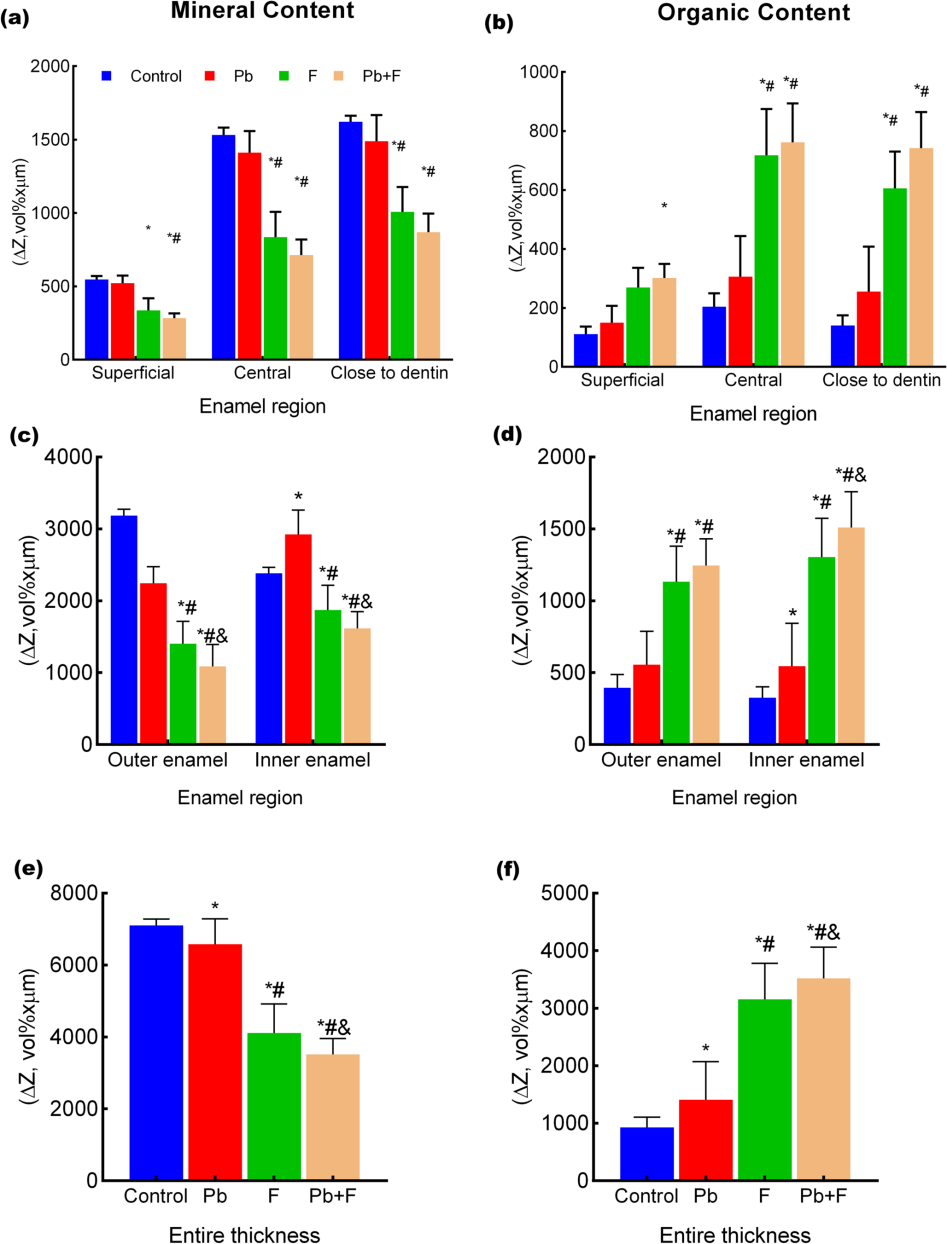
Mineral and organic content across enamel regions. **a**, **c**, **e** Mineral content and **b**, **d**, **f** organic content expressed as ΔZ values (ΔZ, vol%·µm), obtained by integration of the volume–distance profiles across defined enamel regions. Panels (a, b) represent the superficial layer (7–15 μm), central enamel (40–60 μm), and regions close to the dentin–enamel junction (80–100 μm). Panels **c**, **d** show data integrated for the outer enamel (7–40 μm) and inner enamel (60–100 μm), and panels **e**, **f** represent the entire enamel thickness (7–100 μm). Hypomineralization in dentin near the enamel-dentin junction was a frequent finding in the F and Pb + F groups (Table S5). Bars represent mean ± SD. *p < 0.05 versus control; #*p* < 0.05 versus Pb group; &*p* < 0.05 versus F group

The integrated ΔZ values revealed region-dependent compositional alterations in enamel among the experimental groups (Fig. 4). In the entire enamel thickness, fluoride-exposed groups (F and Pb + F) showed a significant reduction in mineral content and an increase in organic content compared with the control, and the Pb group also differed from the control, exhibiting intermediate values. In the half-enamel analysis, the outer enamel displayed only minor differences among groups, whereas the inner enamel concentrated the most pronounced alterations, with greater mineral loss and increased organic content in the F and Pb + F groups.

**Fig. 5:**
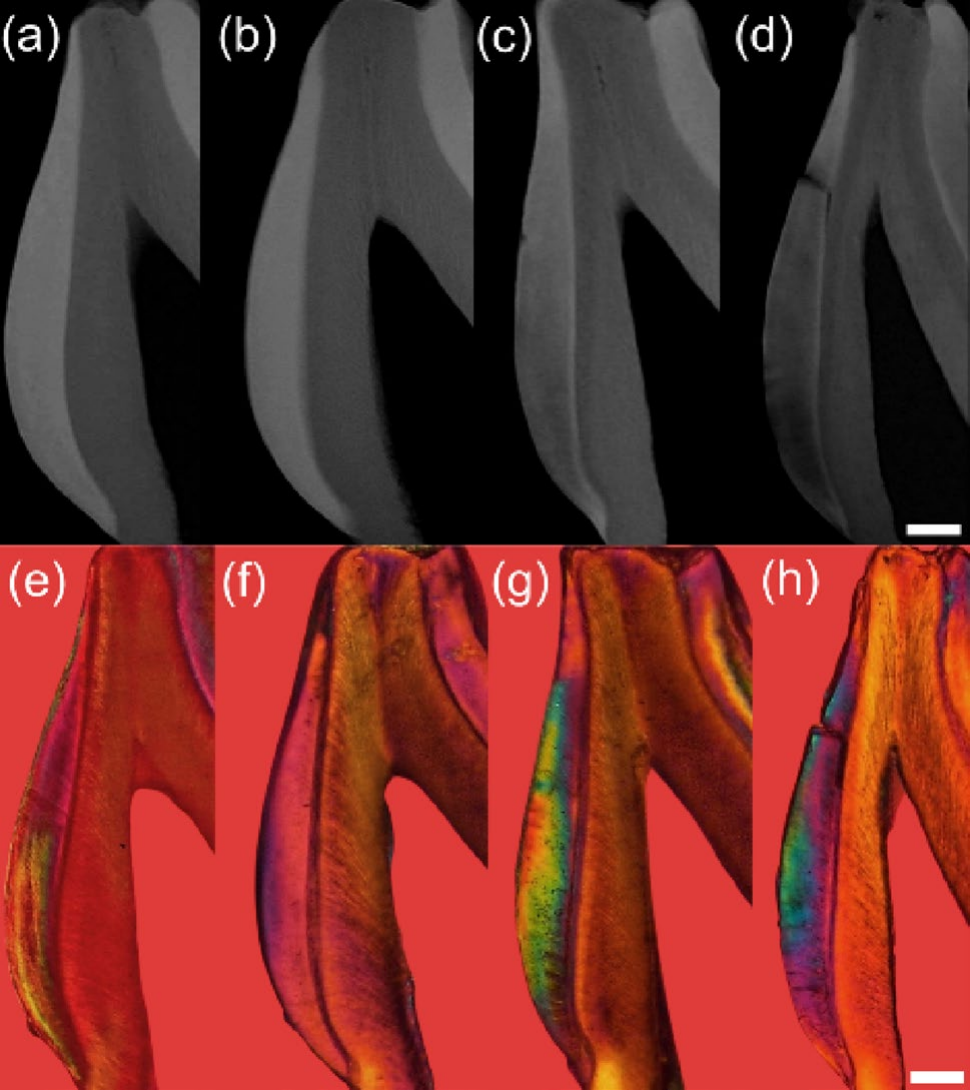
Representative radiomicrographs (**a–d**) and corresponding aspect under polarizing microscopy (e-h) of longitudinal Sects. (80–100 μm thickness) of 30-day-old rat mandibular third molars, showing the mesial surface of the dental enamel. (**a** and **e**) Control group, (**b** and **f**) Pb-exposed group, (**c** and **g**) fluoride-exposed group, and (**d** and **h**) Pb + F co-exposed group. Images show increased radiolucency and more intense positive birefringence (water immersion) in the enamel of the fluoride (**c**) and Pb + F (**d**) groups, indicating the presence of hypomineralized regions when compared with control and Pb-only specimens. Of note, in the Pb + F (**d**) group, the lower part of the enamel is more radiolucent than the dentine of the same tooth. Visually, mineralization in the innermost enamel may appear normal in both control and fluoride-exposure groups due to the optical illusion in the subjective perceived luminance of gray levels [47], while the actual contrast is objectively represented by the effect size in Fig. 6. Scale bar = 100 μm

In the depth-resolved analysis, the superficial layer exhibited the smallest differences in ΔZ values among groups. In the central enamel, significant reductions in mineral ΔZ values and increases in organic ΔZ values were observed in fluoride-containing groups. The regions close to the dentin–enamel junction showed the most marked ΔZ contrasts, particularly in the Pb + F group, characterized by lower mineral content and higher organic content compared with the control and Pb groups. Figure 5 presents representative radiomicrographs showing increased enamel radiolucency in fluoride-treated groups, most pronounced in the central and close to dentin regions; notably, in the Pb + F group, cervical enamel appears qualitatively more radiolucent than the underlying dentin.

**Fig. 6:**
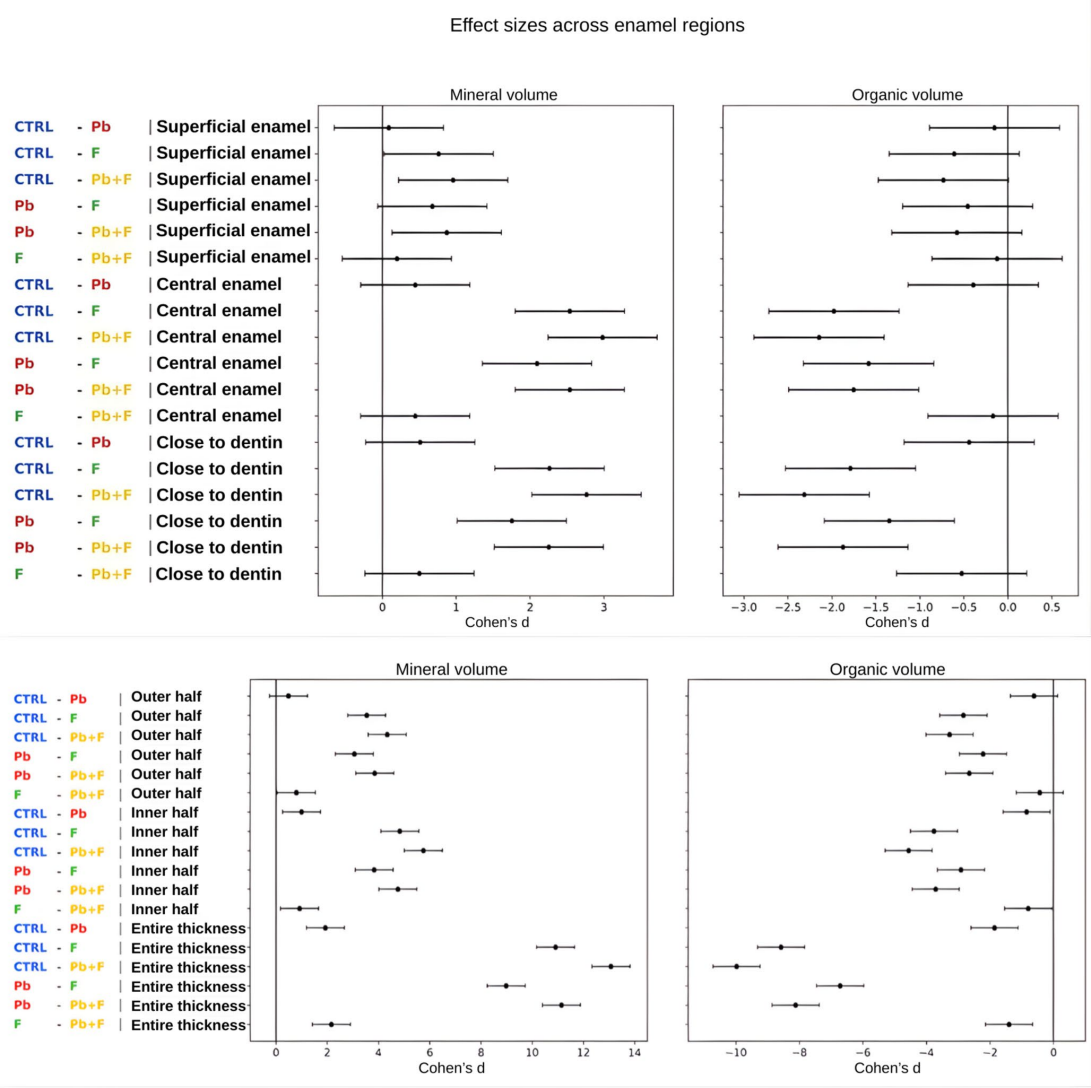
Forest plots showing effect sizes (Cohen’s *d*) and their 95% confidence intervals for pairwise contrasts between exposure groups across different enamel regions. The figure summarizes changes in mineral volume and organic volume evaluated in distinct spatial scales of the enamel. For the region-specific analysis, contrasts are presented for the superficial enamel, central enamel, and regions close to dentin, while the larger-scale analysis includes the outer half, inner half, and entire thickness. Effect sizes represent differences between exposure groups (CTRL, Pb, F, and Pb + F), with positive values indicating higher mineral volume and negative values indicating lower organic volume of the reference group (the first abbreviation in the left corner of each line). For the compared groups (the second abbreviation in the left corner of each line), results indicate hypomineralization (graphs on the left) and organic enrichment (graphs on the right), respectively. The vertical reference line at zero denotes the absence of effect. All estimates were derived from mixed-effects models accounting for treatment-by-region interactions

Forest plots of Cohen’s *d* effect sizes highlighted marked differences in the magnitude of treatment effects across enamel regions for both mineral and organic volumes (Fig. 6). For mineral volume, the largest effect sizes were consistently observed in comparisons involving fluoride exposure, particularly in the central enamel and in regions close to dentin. Contrasts between control and fluoride-containing groups (CTRLxF and CTRLxPb + F) showed large positive effect sizes in these deeper regions, indicating pronounced reductions in mineral volume associated with fluoride exposure. Comparisons between Pb and fluoride-containing groups also yielded moderate-to-large effect sizes in the central enamel and close-to-dentin regions, whereas effect sizes in the superficial region were generally smaller. In contrast, comparisons involving only control and Pb (CTRLxPb) exhibited small or negligible effect sizes across all enamel regions.

For organic volume, the pattern was inverse, with the largest effect sizes again observed in comparisons involving fluoride exposure. Comparisons between control and fluoride-containing groups showed large negative effect sizes, particularly in the central enamel and close-to-dentin regions, reflecting substantial increases in organic volume in fluoride-treated groups. Effect sizes for Pb-related contrasts were consistently smaller, remaining close to zero across enamel regions. Overall, effect sizes tended to increase with depth for fluoride-related contrasts, while the superficial region’s comparisons generally showed the smallest effect sizes for both mineral and organic components.

Regarding Pb and F measurementsin bone, a significant increase in Pb levels was observed in the Pb-exposed groups, particularly under co-exposure (Pb + F), while F concentrations were elevated in the groups receiving fluoride, either alone or in combination (results presented in the Supplementary Material, Figs. S3–S4).

## Discussion

This study showed the importance of spatially-resolved compositional data for evaluating the effect of fluoride and lead on amelogenesis, with specific signature profiles for peak hypomineralization and peak organic enrichment: in the central region for the contrasts in fluoride groups (Control x F and Pb x Pb + F) and in the inner enamel for Pb-containing groups (Control × Pb and F × Pb + F). The smallest effect was located in the superficial region for all groups. These features have important implications for the understanding of the mechanism of impaired enamel maturation due to both fluoride and Pb exposures.

In the present study, control enamel showed a mean mineral content of 89% by weight, consistent with advanced enamel maturation. Similar values were reported by Smith et al. [28] using ash-weight analysis, in which mature wild-type mouse incisor enamel contained ~ 82–89% mineral by dry weight. In contrast, Smith et al. [28] demonstrated that hypomineralized enamel associated with impaired protein removal exhibits substantially reduced mineral fractions (~ 60–75%) and increased organic retention. In agreement with these findings, fluoride-exposed groups in the present study displayed markedly lower mineral content (~ 59–66% by weight) and higher organic matrix (~ 24–28%), closely matching the compositional profile of hypomineralized enamel described by Smith et al. [28]. Despite methodological differences, the close correspondence in mineral and organic weight percentages supports comparable compositional characteristics between the present model and previously described hypomineralized enamel. Furthermore, SEM-EDS-based compositional analysis revealed a progressive decrease in the Ca/C signal ratio across groups (Control > Pb > F > Pb + F) (Table S4). Whether the reported (small) reduction in enamel protein content between 2 and 6 weeks after eruption [29] would affect organic content on our model remains to be determined in future studies.

The more pronounced fluoride-induced hypomineralization within the central region of mature enamel observed in the present study is consistent with the multifactorial mechanisms described in fluorosis and with the functional overlap of enamel proteases during amelogenesis [15]. Classical enzymatic assays demonstrated that fluoride does not directly inhibit KLK4 proteolytic activity [6, 30], indicating preserved catalytic competence; however, in vivo and in vitro studies consistently show reduced KLK4 expression in maturation-stage ameloblasts following fluoride exposure [31, 32]. Experimental models further indicate that MMP20 and KLK4 act sequentially and cooperatively, and that reduced KLK4 availability limits the diffusion and removal of enamel matrix protein fragments from deeper enamel layers [33, 34]. Accordingly, KLK4 deficiency results in a relatively well-mineralized superficial enamel overlying a protein-rich, hypomineralized interior, whereas in MMP20-deficient mice these defects are concentrated near the enamel–dentin junction [15, 33]. Consistent with this pattern, Núñez et al. [35] showed that, in the absence of KLK4, enamel mineralization and hardness are reduced throughout the tissue, with disproportionately greater impairment of the inner and middle enamel layers. On this basis, our findings are consistent with the possibility that impaired enamel maturation caused by F, Pb, and their combination may involve disturbed functional overlap of enamel proteases across the enamel layer, preferentially affecting deep enamel maturation.

In addition, evidence from Aulestia et al. [7] indicates that fluoride exposure alters ameloblast physiology by inducing endoplasmic reticulum stress, modifying intracellular calcium handling, and impairing secretory and transmembrane transport functions. These alterations are accompanied by disturbances in acid–base regulation and bicarbonate transport. Since deep enamel mineralization occurs under spatially constrained conditions that require tight control of the local microenvironment, such cellular alterations may help contextualize the depth-dependent mineralization patterns observed in fluoride-exposed groups, without implying a direct causal relationship.

There are opposing microradiographic results on the presence of a hypermineralized surface layer covering an underlying porous enamel in fluorotic enamel, with positive findings in both human [36] and animal [36, 37] fluorotic enamel and negative findings in human fluorotic enamel [38]. It has been proposed that the formation of a hypermineralized layer would impair proper enamel maturation, but support from quantitative mineral data is lacking. In the present study, irrespective of the contaminant type, the smallest compositional effects were consistently observed in the superficial region, increasing inward. In addition, enamel permeability in the superficial region was never lower than in the other regions, even in the control group, providing no support for the “hypermineralized layer” theory.

In the present study, statistically significant differences between the Pb-containing and other groups (control and fluoride) were shown only in spatially-resolved compositional data, not in fluorosis scores data. This is consistent with the differential compositional profiles of Pb- and F-contaminations, with the former affecting mostly (and with less intensity) inner enamel (not seen upon surface visual examination) and the latter affecting mostly (and with larger intensity) central enamel (seen by translucency upon surface visual examination). Environmental contamination by heavy metals can result in their incorporation into dental tissues during development [10, 11]. Gerlach et al. [12] demonstrated that Pb inhibits enamel matrix proteolytic activity in vitro, likely affecting zinc-dependent enamel proteinases such as MMP-20. The absence of visual hypomineralization in vivo, in the groups exposed only to Pb, highlights the complexity of Pb-related effects on enamel and suggests that Pb exposure alone may not be sufficient to produce detectable structural alterations under the conditions examined.

When fluoride and Pb were combined, however, the present data showed higher fluorosis scores (including enamel loss) and more pronounced compositional disturbances than those observed with fluoride alone. Enamel loss was probably caused by intraoral wear, as impaired maturation does not lead to hypoplasia [15]. These findings suggest that Pb exposure may modulate the severity of fluoride-associated enamel alterations, even though Pb alone produces smaller effects, supporting the importance of considering combined environmental exposures when evaluating developmental enamel defects.

Previous studies demonstrated that lead exacerbates fluoride-induced enamel defects primarily through macroscopic and qualitative analyses. Using rat incisors, Leite et al. [14] reported significantly higher fluorosis scores in animals co-exposed to F (100 ppm) and Pb (30 ppm) compared with fluoride alone, with median scores increasing from ~ 2.0 to ~ 3.25 in upper incisors and from ~ 2.0 to ~ 4.0 in lower incisors, despite similar fluoride concentrations in calcified tissues [14]. While these findings established that lead aggravates fluorosis severity, they did not address how this interaction affects enamel composition or its spatial distribution. In contrast, the present study provides a depth-resolved, quantitative analysis of enamel formed under fluoride and lead co-exposure. Using mandibular third molars, we showed that fluoride markedly reduced mineral volume from ~ 71.0 vol% in controls to ~ 41.1 vol% in the fluoride group and to ~ 35.2 vol% in the Pb + F group, with concomitant increases in organic matrix (~ 9.3 vol% to ~ 31.6 vol% and ~ 35.2 vol%, respectively). Notably, these effects were most pronounced in the central and inner enamel regions.

The mandibular incisors are the teeth most commonly used for quantitative and qualitative analyses of mineral content in rat models of hypomineralization, as their continuous growth enables the investigation of ameloblasts at all stages of differentiation, in addition to allowing clear visualization of hypomineralized lesions. For this reason, these teeth are widely employed in several studies for the identification and characterization of such defects [14, 39]. In the present study, the animals exposed to fluoride exhibited changes in the color of the mandibular incisor teeth (Fig. S5). The dose of fluoride in the water (50 mg/L) was selected from studies in the literature as the lower dose that had been described to cause fluorosis in rat mandibular incisors. And indeed, all animals from this study displayed color changes (often a more opaque, whitish band seen in the incisors). Co-exposure to Pb resulted in discoloration with white and pigmented bands in the incisors. In contrast, exposure to Pb alone did not result in any phenotypic changes, neither in observations by two observers under a stereomicroscope, nor on photographs.

Rat incisors present a superficial iron-rich (Fe) layer [16] (Fig. S6a), which can interfere with X-ray-based measurements of enamel mineral content, as variations in surface Fe affect X-ray absorption. Moreover, mineral volume calculations rely on enamel density values derived from the generic hydroxyapatite formula [21, 22], which does not account for Fe incorporation, potentially leading to inaccurate estimates. In contrast, rat molars lack a superficial iron layer (Fig. S6b) and therefore provide a more reliable model for quantitative assessment of mineral volumes without interference from Fe.

Another factor that limits the use of rodent incisors is the presence of a complex cross-over pattern of enamel prism decussation in these teeth [17, 40, 41], troubling phase-retardation measurements, while rodent molar enamel presents relatively linear prismatic orientation, enabling proper birefringence quantification.

Kurahashi et al. [42] showed that rat third molars develop with enamel maturation beginning around postnatal day 23 and eruption near day 27, rendering them particularly susceptible to environmental contaminants that increase after weaning (day 21). In this context, Hallen [43] demonstrated that offspring from dams continuously exposed to 12 mM Pb-acetate via drinking water exhibited 6-fold higher blood and brain Pb levels when exposure occurred via both placenta and milk compared with placental exposure alone. In contrast, fluoride transfer during nursing is limited. Drinkard et al. [18] reported that dams receiving 0, 50, or 100 ppm F had plasma F levels of 0.02 ± 0.005, 0.10 ± 0.031, and 0.21 ± 0.057 ppm, respectively, with milk concentrations approximately twice those of plasma. Plasma F levels in control pups were 0.003 ± 0.0002 ppm and increased to only 0.006 ± 0.0002 ppm in pups exposed to 100 ppm F; using a mean pup plasma value of 0.0045 ppm, pups in the 50 ppm group had 22-fold lower plasma F than their mothers. This differential exposure during lactation suggests relative protection of pups from high fluoride levels and may explain why fluorosis in young rats is primarily observed in third molars (among molars), whose enamel matures after weaning, when fluoride intake from drinking water (~ 0.2 ppm in the 50 ppm group) becomes approximately 250-fold higher than that from milk.

Pb exposure alone was associated with detectable alterations in amelogenesis only in inner enamel in the present study, which is consistent with the evidence that Pb is a well-characterized inhibitor of delta-aminolevulinic acid dehydratase, an essential enzyme in heme synthesis, and has been shown to induce anemia at doses comparable to those used here, including in rats [44], which may reduce oxygen availability during enamel maturation. Increased fluorosis severity has been reported in populations living at high altitudes, and has been primarily attributed to altered renal acid–base balance and increased fluoride retention [45]; however, chronic low oxygen tension affects a large proportion of high-altitude residents [46], which may represent an additional contributing factor to the mechanism of impaired enamel maturation. Thus, anemia and reduced tissue oxygenation may represent additional speculative factors contributing to the exacerbation of enamel fluorosis.

## Conclusion

In conclusion, fluoride was identified as the primary agent responsible for inducing enamel hypomineralization, particularly in the central and inner enamel regions, while lead co-exposure exacerbated these lesions by affecting mostly inner enamel, highlighting the relevance of combined environmental exposures during enamel development. In addition to validating the mandibular third molar as complementary to rodent incisors, this study demonstrates that this model enables enamel analysis with sufficient spatial resolution to detect depth-dependent alterations associated with disturbed amelogenesis.

## Supplementary Information

Below is the link to the electronic supplementary material.

Supplementary Material 1

## References

[1] Nanci A (2018) Ten Cate’s oral histology development, structure, and function, 9th edn. Elsevier, St. Louis

[2] Mahoney E, Ismail FSM, Kilpatrick N, Swain M (2004) Mechanical properties across hypomineralized/hypoplastic enamel of first permanent molar teeth. Eur J Oral Sci 112:497–502. 10.1111/j.1600-0722.2004.00162.x 15560832 10.1111/j.1600-0722.2004.00162.x

[3] Wright JT (2023) Enamel phenotypes: genetic and environmental determinants. Genes (Basel) 14:545. 10.3390/GENES14030545 36980818 10.3390/genes14030545 PMC10048525

[4] Aoba T, Fejerskov O (2002) Dental fluorosis: chemistry and biology. Crit Rev Oral Biol Med 13:155–170. 10.1177/154411130201300206 12097358 10.1177/154411130201300206

[5] DenBesten PK, Crenshaw MA (1987) Studies on the changes in developing enamel caused by ingestion of high levels of fluoride in the rat. Adv Dent Res 1:176–180. 10.1177/08959374870010020501;CTYPE:STRING:JOURNAL 3504167 10.1177/08959374870010020501

[6] Gerlach RF, Souza AP, Cury JA, Line SRP (2000) Fluoride effect on the activity of enamel matrix proteinases in vitro. Eur J Oral Sci 108:48–53 10706477 10.1034/j.1600-0722.2000.00735.x

[7] Aulestia FJ, Groeling J, Bomfim GHS et al (2020) Fluoride exposure alters Ca^2+^ signaling and mitochondrial function in enamel cells. Sci Signal 13:86. 10.1126/SCISIGNAL.AAY0086 ;ISSUE:ISSUE:DOI 10.1126/scisignal.aay0086 PMC7173621 32071168

[8] Watson GE, Davis BA, Raubertas RF et al (1997) Influence of maternal lead ingestion on caries in rat pups. Nat Med 1997 3:9. 10.1038/nm0997-1024 10.1038/nm0997-1024 9288731

[9] Gerlach RF, Cury JA, Krug FJ, Line SRP (2002) Effect of lead on dental enamel formation. Toxicology 175:27–34. 10.1016/S0300-483X(02)00082-3 12049833 10.1016/s0300-483x(02)00082-3

[10] de Almeida GRC, Pereira Saraiva M, da Barbosa C F, et al (2007) Lead contents in the surface enamel of deciduous teeth sampled in vivo from children in uncontaminated and in lead-contaminated areas. Environ Res 104:337–345. 10.1016/j.envres.2007.03.007 17512519 10.1016/j.envres.2007.03.007

[11] de Souza-Guerra C, Barroso RC, de Almeida AP et al (2014)Anatomical variations in primary teeth microelements with known differences in lead content by micro-synchrotron radiation X-ray fluorescence (µ-SRXRF)—a preliminary study. J Trace Elem Med Biol 28:186–193. 10.1016/j.jtemb.2014.01.007 24656317 10.1016/j.jtemb.2014.01.007

[12] Gerlach RF, De Souza AP, Cury JA, Line SRP (2000) Effect of lead, cadmium and zinc on the activity of enamel matrix proteinases in vitro. Eur J Oral Sci 108:327–334. 10.1034/J.1600-0722.2000.108004327.X 10946768 10.1034/j.1600-0722.2000.108004327.x

[13] Sawan RMM, Leite GAS, Saraiva MCP et al (2010) Fluoride increases lead concentrations in whole blood and in calcified tissues from lead-exposed rats. Toxicology 271:21–26. 10.1016/j.tox.2010.02.002 20188782 10.1016/j.tox.2010.02.002

[14] Leite GAS, Sawan RMM, Teófilo JM et al (2011) Exposure to lead exacerbates dental fluorosis. Arch Oral Biol 56:695–702. 10.1016/j.archoralbio.2010.12.011 21269604 10.1016/j.archoralbio.2010.12.011

[15] Yamakoshi Y, Richardson AS, Nunez SM et al (2011) Enamel proteins and proteases in Mmp20 and Klk4 null and double-null mice. Eur J Oral Sci 119:206–216. 10.1111/j.1600-0722.2011.00866.x 22243248 10.1111/j.1600-0722.2011.00866.x PMC3282035

[16] Srot V, Houari S, Kapun G et al (2024) Ingenious architecture and coloration generation in enamel of rodent teeth. ACS Nano 18:11270–11283. 10.1021/acsnano.4c00578 38629732 10.1021/acsnano.4c00578 PMC11064225

[17] Risnes S (1979) The prism pattern of rat molar enamel: a scanning electron microscope study. Am J Anat 155:245–257. 10.1002/aja.1001550207 474447 10.1002/aja.1001550207

[18] Drinkard CR, Deaton TG, Bawden JW (1985) Enamel fluoride in nursing rats with mothers drinking water with high fluoride concentrations. J Dent Res 64:877–880. 10.1177/00220345850640060301 3858313 10.1177/00220345850640060301

[19] Thylstrup A, Fejerskov O (1978) Clinical appearance of dental fluorosis in permanent teeth in relation to histologic changes. Commun Dent Oral Epidemiol 6:315–328.10.1111/J.1600-0528.1978.TB01173.X 10.1111/j.1600-0528.1978.tb01173.x 282114

[20] Dantas ELD, de Figueiredo A, Macedo-Ribeiro JT N, et al (2020) Variation in mineral, organic, and water volumes at the neonatal line and in pre- and postnatal enamel. Arch Oral Biol 118:104850. 10.1016/j.archoralbio.2020.104850 32736142 10.1016/j.archoralbio.2020.104850

[21] Elliott JC (1997) Structure, crystal chemistry and density of enamel apatites. CIBA Found Symp. 10.1002/9780470515303.ch5 9189617

[22] Angmar B, Carlström D, Glas JE (1963) Studies on the ultrastructure of dental enamel. IV. The mineralization of normal human enamel. J Ultrasructure Res 8:12–23. 10.1016/S0022-5320(63)80017-9 10.1016/s0022-5320(63)80017-9 14013184

[23] Gan HY, Sousa FB, Carlo HL et al (2015) Enhanced transport of materials into enamel nanopores via electrokinetic flow. J Dent Res 94:615–621. 10.1177/0022034515572189;WGROUP:STRING:PUBLICATION 25691072 10.1177/0022034515572189

[24] Sousa FB, Vianna SS, Santos-Magalhães NS (2006) A new approach for improving the birefringence analysis of dental enamel mineral content using polarizing microscopy. J Microsc 221:79–83. 10.1111/J.1365-2818.200601547.X;CTYPE:STRING:JOURNAL 16499547 10.1111/j.1365-2818.2006.01547.x

[25] De Sousa FB, Soares JD, Vianna SS (2013) Natural enamel caries: a comparative histological study on biochemical volumes. Caries Res 47:183–192. 10.1159/000345378 23222001 10.1159/000345378

[26] Rothman KJ (1990) no adjustments are needed for multiple comparisons. Epidemiology 1:43–46 2081237

[27] de Abreu NMR, dos Santos Oliveira R, de Sousa FB (2025) Spatially resolved composition of bovine dental enamel in permanent incisors. Arch Oral Biol. 10.1016/j.archoralbio.2025.106215 40068389 10.1016/j.archoralbio.2025.106215

[28] Smith CE, Hu Y, Richardson AS et al (2011) Relationships between protein and mineral during enamel development in normal and genetically altered mice. Eur J Oral Sci 119:125–135. 10.1111/j.1600-0722.2011.00871.x 22243238 10.1111/j.1600-0722.2011.00871.x PMC3295546

[29] Karaaslan H, Walker AR, Gil-Bona A et al (2025) Posteruptive loss of proteins in porcine enamel. J Dent Res 104:290–298. 10.1177/00220345241299382 39725879 10.1177/00220345241299382 PMC13331254

[30] Tye CE, Antone JV, Bartlett JD (2011) Fluoride does not inhibit enamel protease activity. J Dent Res 90:489. 10.1177/0022034510390043 21118795 10.1177/0022034510390043 PMC3077535

[31] Suzuki M, Shin M, Simmer JP, Bartlett JD (2014) Fluoride affects enamel protein content via TGF-β1-mediated KLK4 inhibition. J Dent Res 93:1022. 10.1177/0022034514545629 25074495 10.1177/0022034514545629 PMC4212320

[32] Le MH, Nakano Y, Uyghurturk DA et al (2017) Fluoride alters Klk4 expression in maturation ameloblasts through androgen and progesterone receptor signaling. Front Physiol 8:276791. 10.3389/FPHYS.2017.00925/BIBTEX 10.3389/fphys.2017.00925 PMC5715335 29249975

[33] Smith CE, Richardson AS, Hu Y et al (2011) Effect of kallikrein 4 loss on enamel mineralization: comparison with mice lacking matrix metalloproteinase 20. J Biol Chem 286:18149–18160. 10.1074/jbc.M110.194258 21454549 10.1074/jbc.M110.194258 PMC3093887

[34] Smith CEL, Kirkham J, Day PF et al (2017) A fourth KLK4 mutation is associated with enamel hypomineralisation and structural abnormalities. Front Physiol. 10.3389/fphys.2017.00333 28611678 10.3389/fphys.2017.00333 PMC5447068

[35] Núñez SM, Chun YHP, Ganss B et al (2016) Maturation stage enamel malformations in Amtn and Klk4 null mice. Matrix Biol 52–54:219–233 26620968 10.1016/j.matbio.2015.11.007 PMC4875837

[36] Fejerskov O, Yaeger JA, Thylstrup A (1979) Microradiography of the effect of acute and chronic administration of fluoride on human and rat dentine and enamel. Arch Oral Biol 24:123–130 10.1016/0003-9969(79)90060-8 299137

[37] Lyaruu DM, Medina JF, Sarvide S et al (2014) Barrier formation: potential molecular mechanism of enamel fluorosis. J Dent Res 93:96–102. 10.1177/0022034513510944 24170372 10.1177/0022034513510944 PMC3865793

[38] de Sousa FB, Pires AC, Figueiredo RCBQ et al (2017) Quantitative study of the proportion of the pore volume of human fluorotic enamel filled by resin infiltrant. Arch Oral Biol 82:134–140. 10.1016/J.ARCHORALBIO.2017.06.017 28641179 10.1016/j.archoralbio.2017.06.017

[39] Jedeon K, Houari S, Loiodice S et al (2016) Chronic exposure to bisphenol A exacerbates dental fluorosis in growing rats. J Bone Miner Res 31:1955–1966. 10.1002/jbmr.2879 27257137 10.1002/jbmr.2879

[40] Lyngstadaas SP, Møinichen CB, Risnes S (1998) Crown morphology, enamel distribution, and enamel structure in mouse molars. Anat Rec 250(199803):268–280. 10.1002/(SICI)1097-0185 9517844 10.1002/(SICI)1097-0185(199803)250:3<268::AID-AR2>3.0.CO;2-X

[41] Pessoa-Lima C, Tostes-Figueiredo J, Macedo-Ribeiro N et al (2022) Structure and chemical composition of ca. 10-million-year-old (late Miocene of Western Amazon) and present-day teeth of related species. Biology (Basel). 10.3390/BIOLOGY11111636 36358337 10.3390/biology11111636 PMC9687460

[42] Kurahashi Y, Nagai N, Watanabe K et al (1968) Chronological observation of the odontogenesis of rat molars (1). Bull Tokyo dent coll 9:147–159 5250597

[43] Hallén IP, Jorhem L, Oskarsson A (1995) Placental and lactational transfer of lead in rats: a study on the lactational process and effects on offspring. Arch Toxicol 1995 69(9 69):596–602. 10.1007/S002040050219 10.1007/s002040050219 8660136

[44] Jang WH, Lim KM, Kim K et al (2011) Low level of lead can induce phosphatidylserine exposure and erythrophagocytosis: a newmechanism underlying lead-associated anemia. Toxicol Sci 122:177–184. 10.1093/TOXSCI/KFR079 21482638 10.1093/toxsci/kfr079

[45] Angmar-Mansson B, Whitford GM (1990) Environmental and physiological factors affecting dental fluorosis. J Dent Res. 10.1177/00220345900690S137 2179333 10.1177/00220345900690S137

[46] Soria R, Egger M, Scherrer U et al (2016) Pulmonary artery pressure and arterial oxygen saturation in people living at high or low altitude: systematic review and meta-analysis. J Appl Physiol (1985) 121:1151–1159. 10.1152/JAPPLPHYSIOL.00394.2016 27660297 10.1152/japplphysiol.00394.2016

[47] Adelson EH (1993) Perceptual organization and the judgment of brightness. Science 262:2042–2044. 10.1126/science.8266102 8266102 10.1126/science.8266102

