# Supplementary material for: Effects of fluoride and lead on enamel composition during the maturation stage of amelogenesis in rat mandibular third molars
Source: Calcif Tissue Int. 2026 Apr 2;117(1):52. doi: 10.1007/s00223-026-01511-z (PMC13046661; doi:10.1007/s00223-026-01511-z)
Supplement: Supplementary file 1 — Supplementary Material 1 [file 223_2026_1511_MOESM1_ESM.docx]

**Effects of Fluoride and Lead on Enamel Composition During the Maturation Stage of Amelogenesis in Rat Mandibular Third Molars**

[Calcified Tissue International](https://link.springer.com/journal/223)

**Materials and Methods**

###

**Criteria applied for selecting the tooth and the tooth surface**

### The following criteria were used to select the most suitable tooth surface: (i) the presence of iron-free enamel; (ii) predominance of parallel prisms; (iii) ability to undergo mechanical wear without enamel delamination in the control group; (iv) a relatively homogenous morphological phenotype; and (v) to undergo enamel maturation after weaning. Only the mesial surface of the third molar of 30-day-old mice fulfilled all requirements.

### Scanning Electron Microscopy Coupled with Energy-Dispersive X-ray Spectroscopy (SEM–EDS)

The microstructural and elemental analysis of dental enamel was performed using scanning electron microscopy coupled with energy-dispersive X-ray spectroscopy (SEM–EDS), employing a JSM-6610LV microscope (JEOL, Tokyo, Japan) with a nominal resolution of 3 nm. Analyses were conducted under low-vacuum conditions, using a backscattered electron (BSE) detector, without prior coating of the samples with gold or carbon. EDS data acquisition and processing were carried out using the AZtec software (Oxford Instruments, Oxford, UK), with elemental signals recorded as counts per second (cps). SEM–EDS was applied to identify and map the chemical elements present in dental enamel.

Two analytical approaches were employed. First, a comparative surface analysis between incisors and molars was performed through elemental mapping of the enamel surface, with particular emphasis on assessing differences in iron presence between the two dental groups. Second, linear scans (line scans) were conducted on molar cross-sections, covering the entire enamel thickness from the outer surface to the enamel–dentin junction, with the specific aim of mapping calcium and carbon distributions within the enamel layer of each experimental group. Mean semiquantitative values of calcium and carbon, expressed as counts per second (cps), as well as the Ca/C ratio, were calculated to provide an estimate of the relative proportions of mineralized and organic components among groups [1]. Carbon concentration should be interpreted with caution, as it may originate from carbonate and organic matter. These values for calcium cps, carbon cps, and the corresponding Ca/C ratios are reported in Table S3 (n = 3 per group).

**Enamel permeability**

Enamel permeability was measured from the water (a) and non-mineral (the sum of the organic and water volumes, V_2_) volumes, using the following equation [2]:

Permeability = a² / V₂

As a reference, the permeability of sound mature human permanent enamel was reported to be 4.15 (± 0.81) [2]. Descriptive statistical data on enamel permeability per histological point of interest are shown in Supplementary Table S1. Results on the descriptive statistics and inferential pairwise paired T tests on intragroup contrasts of the AUC on the enamel permeability among enamel regions are shown in Supplementary Table S2. AUC of the superficial region was adjusted by multiplying it by 2.5, which is the correction factor for its smaller area relative to other regions.

**Results**

**
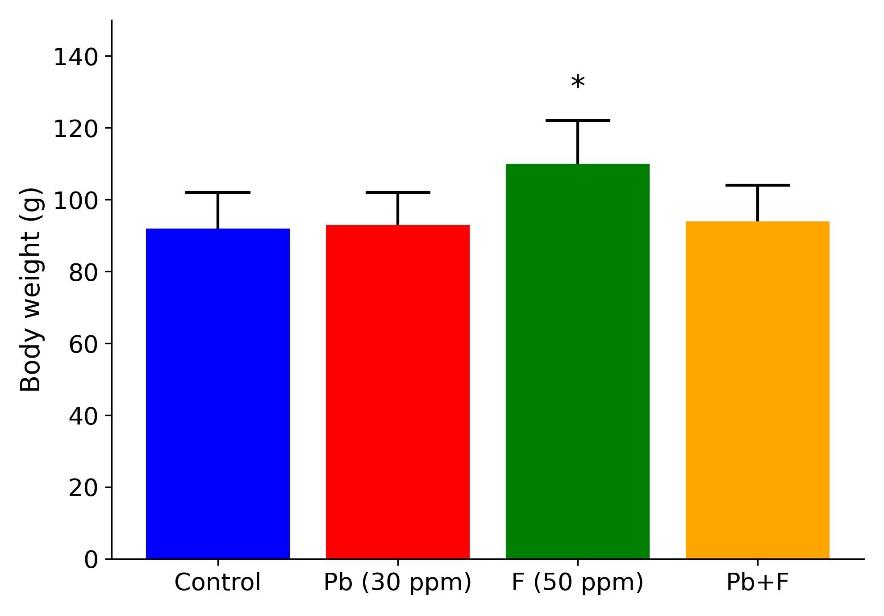
**

**Fig. S1** Body weight (mean ± SD) of 30-day-old rats from the Control, Pb (30 ppm), F (50 ppm), and Pb+F groups at euthanasia. Animals exposed only to fluoride showed a significantly higher body weight compared with the Control group (*p < 0.05), whereas no significant differences were observed among the other groups. Although fluoride exposure has been associated with alterations in certain metabolic parameters, there is no consistent evidence that fluoride induces weight gain in animal models [3-4]


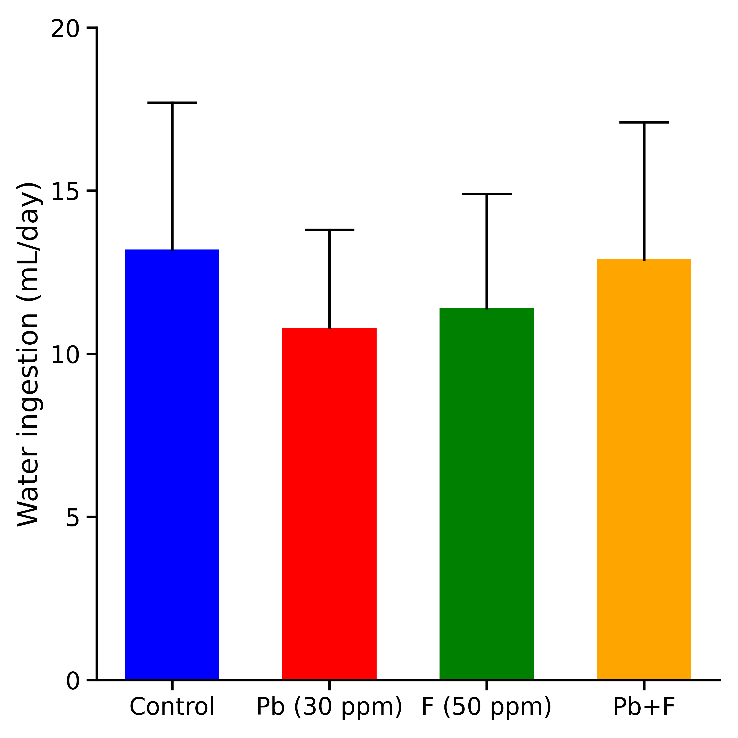


**Fig. S2** Water intake (mean ± SD) of 30-day-old rats from the Control, Pb (30 ppm), F (50 ppm), and Pb+F groups during the experimental period. No statistically significant differences were observed among the groups (p > 0.05). Monitoring water consumption was essential to ensure that the use of fluorosilicic acid did not alter water palatability to the extent of affecting intake. Fluorosilicic acid is one of the most widely used compounds for community water fluoridation worldwide [5-7].


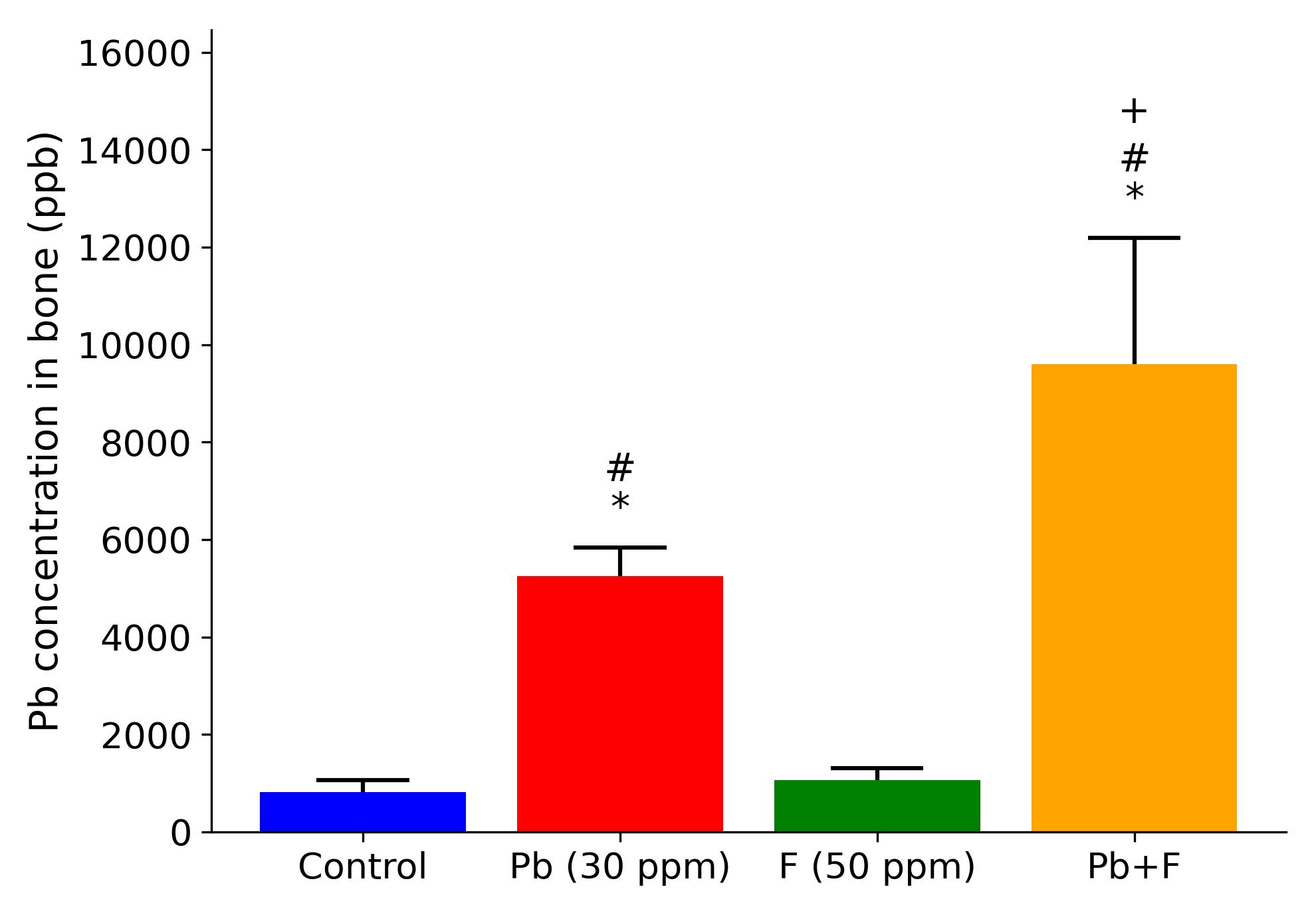


**Fig. S3** Lead (Pb) concentration in bone (ppb) of 30-day-old rats from the Control, Pb (30 ppm), F (50 ppm), and Pb+F groups, determined by graphite furnace atomic absorption spectrometry (GFAAS). Bars represent mean values, and upper error bars indicate standard deviation. * indicates a significant difference compared with the Control group (*p<0.0001); # indicates a significant difference compared with the F group (#p < 0.0001); + indicates a significant difference compared with the Pb group (+p< 0.0001)


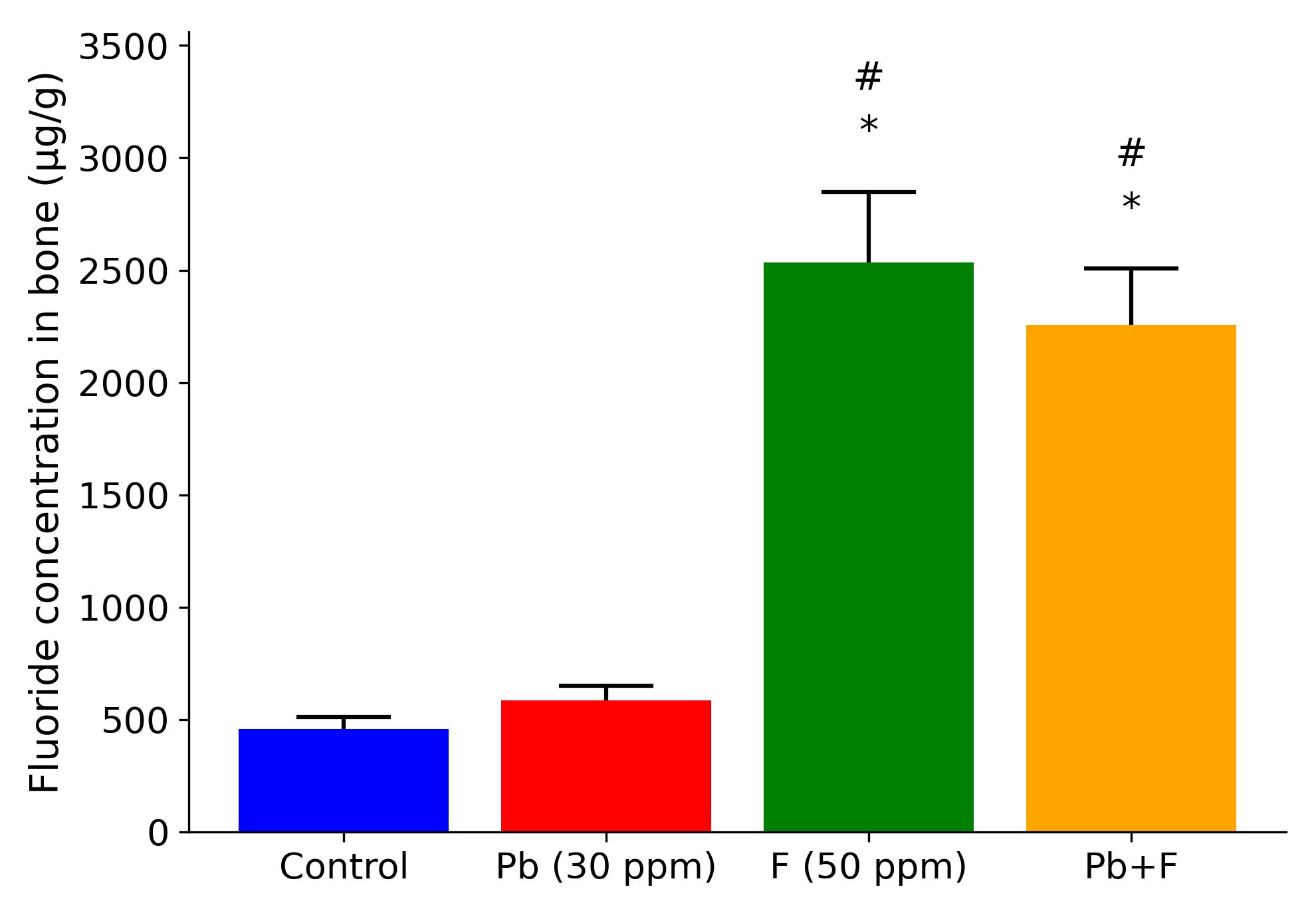


**Fig. S4** Fluoride concentration in bone (µg/g) of 30-day-old rats from the Control, Pb (30 ppm), F (50 ppm), and Pb+F groups, determined using a fluoride ion-selective electrode (F-ISE). Bars represent mean values, and upper error bars indicate standard deviation. * indicates a significant difference compared with the Control group (*p < 0.0001); # indicates a significant difference compared with the Pb group (#p < 0.0001)


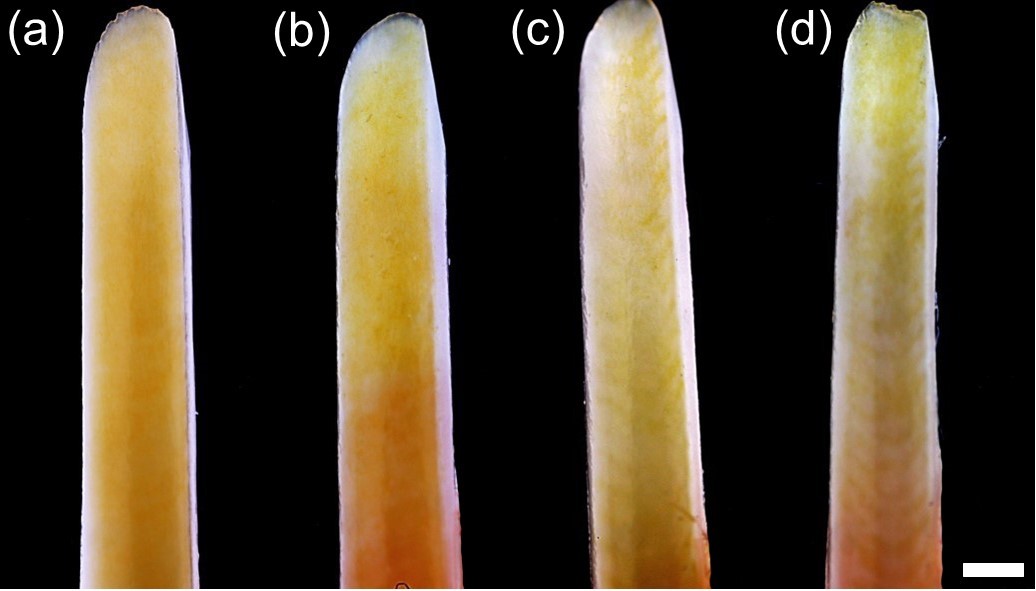


**Fig. S5** Representative stereomicroscopic images of mandibular incisors from rats in the different experimental groups. (a) Control group (water), showing predominantly yellow to orange coloration, with no visible signs of dental fluorosis. (b) Lead-exposed group (Pb), displaying a chromatic pattern similar to the control, without evidence of white or brown bands associated with fluorosis. (c) Fluoride-exposed group (F), exhibiting evident signs of dental fluorosis, characterized by partial loss of pigmentation and the presence of whitish bands and brownish areas along the enamel surface. (d) The co-exposure group to lead and fluoride (Pb+F) presented the most pronounced alterations, with marked discoloration, more intense white and brown bands, and greater impairment of enamel pigmentation. Collectively, the images indicate that co-exposure exacerbates enamel surface alterations compared with single-contaminant exposure, even under conditions of lower dose and shorter exposure duration. Bar = 500 μm


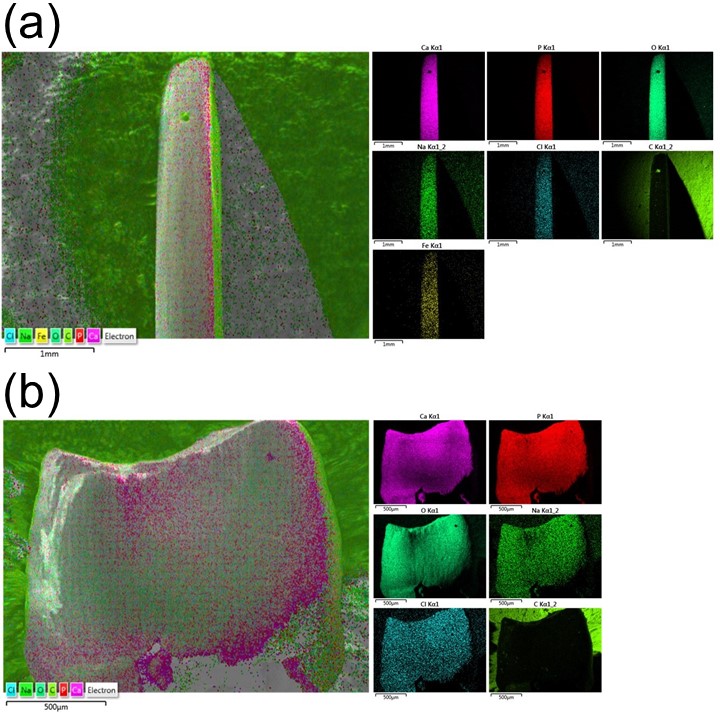


**Fig. S6** Representative SEM–EDS elemental maps of enamel surfaces from control animals. (a) Elemental mapping of the enamel surface of mandibular incisors from the control group. (b) Elemental mapping of the enamel surface of mandibular third molars from the control group. The same major elements were detected on the enamel surface of both dental types, including calcium, phosphorus, oxygen, sodium, chlorine, and carbon. Notably, iron was detected exclusively on the enamel surface of incisors, whereas it was absent from the enamel surface of third molars

**Table S1** Descriptive statistics on enamel permeability as a function of the distance from the enamel surface per group (n = 10).

| Distance from enamel surface | Mean | SD |
| --- | --- | --- |
| *Control group* |  |  |
| 7 | 10.69 | 2.09 |
| 15 | 9.60 | 2.09 |
| 40 | 7.80 | 0.89 |
| 60 | 7.51 | 0.60 |
| 80 | 7.45 | 0.49 |
| 100 | 7.66 | 0.38 |
|  |  |  |
| *Pb group* |  |  |
| 7 | 7.64 | 3.04 |
| 15 | 7.68 | 3.00 |
| 40 | 7.10 | 1.56 |
| 60 | 6.90 | 1.52 |
| 80 | 6.77 | 1.10 |
| 100 | 6.72 | 0.88 |
|  |  |  |
| *F group* |  |  |
| 7 | 11.79 | 7.30 |
| 15 | 10.21 | 4.87 |
| 40 | 9.08 | 4.35 |
| 60 | 9.34 | 4.45 |
| 80 | 8.28 | 3.01 |
| 100 | 6.97 | 1.21 |
|  |  |  |
| *Pb+F group* |  |  |
| 7 | 12.60 | 5.24 |
| 15 | 10.94 | 4.27 |
| 40 | 11.14 | 4.12 |
| 60 | 11.29 | 4.61 |
| 80 | 8.27 | 2.92 |
| 100 | 5.55 | 1.34 |

**Table S2** Descriptive statistics and inferential statistics on paired contrasts of the AUC on the enamel permeability among enamel regions within groups.

| Distance from enamel surface | Mean | SD |
| --- | --- | --- |
| *Descriptive statistics* |  |  |
| *Superficial region* |  |  |
| Control | 202.89 | 37.99 |
| Pb | 153.20 | 59.98 |
| F | 220.01 | 119.77 |
| Pb+F | 235.35 | 92.57 |
|  |  |  |
| *Central region* |  |  |
| Control | 153.11 | 14.44 |
| Pb | 140.00 | 30.62 |
| F | 184.27 | 75.64 |
| Pb+F | 224.26 | 85.32 |
|  |  |  |
| *Close to dentin* |  |  |
| Control | 151.15 | 6.42 |
| Pb | 134.87 | 19.12 |
| F | 152.53 | 39.77 |
| Pb+F | 138.19 | 34.04 |
|  |  |  |
| *Inferential statistics* |  |  |
|  | P value | Cohen`s d (CI 95%) |
| *Control group* |  |  |
| Superficial x central | 4.71E-04 | 1.69 (0.51; 2.87) |
| Superficial x close to dentin | 1.81E-03 | 1.38 (0.25; 2.51) |
| Central x close to dentin | 6.868E-01 | 0.13 (-0.88; 1.14) |
|  |  |  |
| *Pb group* |  |  |
| Superficial x central | 2.086E-01 | 0.43 (-0.59; 1.45) |
| Superficial x close to dentin | 2.57E-01 | 0.38 (-0.64; 1.40) |
| Central x close to dentin | 3.862E-01 | 0.29 (-0.73; 1.30) |
|  |  |  |
| *F group* |  |  |
| Superficial x central | 3.496E-01 | 0.31 (-0.71; 1.33) |
| Superficial x close to dentin | 6.83E-02 | 0.65 (-0.38; 1.69) |
| Central x close to dentin | 2.711E-01 | 0.37 (-0.65; 1.39) |
|  |  |  |
| *Pb+F group* |  |  |
| Superficial x central | 6.311E-01 | 0.16 (-0.86; 1.17) |
| Superficial x close to dentin | 6.03E-03 | 1.13 (0.04; 2.22) |
| Central x close to dentin | 1.647E-03 | 1.40 (0.27; 2.53) |

**Table S3.** Component volumes of dental enamel.

## Outcome: AUC_Volume Mineral ANOVA – mixed model AUC_VMIN ~ treatment * region + (1 | ID2)

|  | **Factor** | **Sum Sq** | **Mean Sq** | **NumDF** | **DenDF** | **F value** | **p, value** |
| --- | --- | --- | --- | --- | --- | --- | --- |
|  | Exposure x region | 50,952.706 | 3,396.847 | 15 | 207 | 44.95002 | 1.15537E-56 |

### Contrasts between treatments by region (differences, 95% CI, and Cohen's d)

| **contrast** | **region** | **p, value** | **cohen_d** | **lower, CL_d** | **upper, CL_d** |
| --- | --- | --- | --- | --- | --- |
| CTRL - PB | Entire thickness | 1.277E-05 | 1.92619733 | 1.18728952 | 2.6651051 |
| CTRL - F | Entire thickness | 4.261E-63 | 10.90836733 | 10.16945952 | 11.6472751 |
| CTRL - PB+F | Entire thickness | 1.083E-75 | 13.06826263 | 12.32935482 | 13.8071704 |
| PB - F | Entire thickness | 8.382E-51 | 8.98217000 | 8.24326219 | 9.7210778 |
| PB - PB+F | Entire thickness | 1.606E-64 | 11.14206530 | 10.40315749 | 11.8809731 |
| F - PB+F | Entire thickness | 1.328E-06 | 2.15989530 | 1.42098750 | 2.8988031 |
| CTRL - PB | Outer half | 1.394E-01 | 0.48558916 | -0.25331865 | 1.2244970 |
| CTRL - F | Outer half | 7.110E-14 | 3.54242934 | 2.80352153 | 4.2813371 |
| CTRL - PB+F | Outer half | 7.211E-19 | 4.33650461 | 3.59759680 | 5.0754124 |
| PB - F | Outer half | 4.498E-11 | 3.05684018 | 2.31793238 | 3.7957480 |
| PB - PB+F | Outer half | 9.213E-16 | 3.85091545 | 3.11200765 | 4.5898233 |
| F - PB+F | Outer half | 3.863E-02 | 0.79407527 | 0.05516746 | 1.5329831 |
| CTRL - PB | Superficial enamel | 4.237E-01 | 0.08613425 | -0.65277356 | 0.8250421 |
| CTRL - F | Superficial enamel | 4.491E-02 | 0.76220517 | 0.02329737 | 1.5011130 |
| CTRL - PB+F | Superficial enamel | 1.666E-02 | 0.95816299 | 0.21925519 | 1.6970708 |
| PB - F | Superficial enamel | 6.606E-02 | 0.67607093 | -0.06283688 | 1.4149787 |
| PB - PB+F | Superficial enamel | 2.627E-02 | 0.87202875 | 0.13312094 | 1.6109366 |
| F - PB+F | Superficial enamel | 3.309E-01 | 0.19595782 | -0.54294999 | 0.9348656 |
| CTRL - PB | Central | 1.606E-01 | 0.44474066 | -0.29416715 | 1.1836485 |
| CTRL - F | Central | 2.292E-08 | 2.53901097 | 1.80010317 | 3.2779188 |
| CTRL - PB+F | Central | 1.139E-10 | 2.98332674 | 2.24441893 | 3.7222345 |
| PB - F | Central | 2.557E-06 | 2.09427031 | 1.35536251 | 2.8331781 |
| PB - PB+F | Central | 2.303E-08 | 2.53858608 | 1.79967827 | 3.2774939 |
| F - PB+F | Central | 1.608E-01 | 0.44431576 | -0.29459204 | 1.1832236 |
| CTRL - PB | Inner half | 1.352E-02 | 0.99586751 | 0.25695970 | 1.7347753 |
| CTRL - F | Inner half | 3.909E-22 | 4.82692701 | 4.08801920 | 5.5658348 |
| CTRL - PB+F | Inner half | 1.742E-28 | 5.74843128 | 5.00952347 | 6.4873391 |
| PB - F | Inner half | 1.225E-15 | 3.83105950 | 3.09215169 | 4.5699673 |
| PB - PB+F | Inner half | 1.241E-21 | 4.75256377 | 4.01365596 | 5.4914716 |
| F - PB+F | Inner half | 2.030E-02 | 0.92150427 | 0.18259646 | 1.6604121 |
| CTRL - PB | Close to dentin | 1.270E-01 | 0.51149997 | -0.22740784 | 1.2504078 |
| CTRL - F | Close to dentin | 4.547E-07 | 2.26434286 | 1.52543505 | 3.0032507 |
| CTRL - PB+F | Close to dentin | 1.646E-09 | 2.76535013 | 2.02644232 | 3.5042579 |
| PB - F | Close to dentin | 6.030E-05 | 1.75284289 | 1.01393508 | 2.4917507 |
| PB - PB+F | Close to dentin | 5.072E-07 | 2.25385015 | 1.51494235 | 2.9927580 |
| F - PB+F | Close to dentin | 1.319E-01 | 0.50100727 | -0.23790054 | 1.2399151 |

## Outcome: AUC_VORG ANOVA – mixed model AUC_VORG ~ treatment * region + (1 | ID2)

| **Factor** | **Sum Sq** | **Mean Sq** | **NumDF** | **DenDF** | **F value** | **p value** |
| --- | --- | --- | --- | --- | --- | --- |
| Exposure | 42,839.336 | 14,279.779 | 3 | 207 | 211.60401 | 8.79123E-63 |
| Exposure x region | 26,256.483 | 1,750.432 | 15 | 207 | 25.93867 | 1.56401E-39 |

### Contrasts between treatments by region (differences, 95% CI, and Cohen's d)

| **contrast** | **region** | **p value** | **cohen_d** | **lower,CL_d** | **upper,CL_d** |
| --- | --- | --- | --- | --- | --- |
| CTRL - PB | Entire thickness | 2.371E-05 | -1.8585615 | -2.5974693 | -1.119653679 |
| CTRL - F | Entire thickness | 4.298E-48 | -8.5763967 | -9.3153045 | -7.837488871 |
| CTRL - PB+F | Entire thickness | 2.819E-57 | -9.9772108 | -10.7161186 | -9.238303034 |
| PB - F | Entire thickness | 2.786E-35 | -6.7178352 | -7.4567430 | -5.978927385 |
| PB - PB+F | Entire thickness | 5.422E-45 | -8.1186494 | -8.8575572 | -7.379741548 |
| F - PB+F | Entire thickness | 9.927E-04 | -1.4008142 | -2.1397220 | -0.661906356 |
| CTRL - PB | Outer half | 8.463E-02 | -0.6168908 | -1.3557986 | 0.122017002 |
| CTRL - F | Outer half | 6.944E-10 | -2.8369112 | -3.5758190 | -2.098003435 |
| CTRL - PB+F | Outer half | 2.757E-12 | -3.2719303 | -4.0108381 | -2.533022473 |
| PB - F | Outer half | 7.198E-07 | -2.2200204 | -2.9589282 | -1.481112629 |
| PB - PB+F | Outer half | 6.051E-09 | -2.6550395 | -3.3939473 | -1.916131667 |
| F - PB+F | Outer half | 1.659E-01 | -0.4350190 | -1.1739268 | 0.303888769 |
| CTRL - PB | Superficial enamel | 3.667E-01 | -0.1525694 | -0.8914772 | 0.586338435 |
| CTRL - F | Superficial enamel | 8.708E-02 | -0.6098471 | -1.3487549 | 0.129060689 |
| CTRL - PB+F | Superficial enamel | 5.140E-02 | -0.7328467 | -1.4717545 | 0.006061116 |
| PB - F | Superficial enamel | 1.539E-01 | -0.4572777 | -1.1961856 | 0.281630062 |
| PB - PB+F | Superficial enamel | 9.794E-02 | -0.5802773 | -1.3191851 | 0.158630488 |
| F - PB+F | Superficial enamel | 3.918E-01 | -0.1229996 | -0.8619074 | 0.615908234 |
| CTRL - PB | Central | 1.901E-01 | -0.3932405 | -1.1321483 | 0.345667266 |
| CTRL - F | Central | 7.968E-06 | -1.9765817 | -2.7154895 | -1.237673844 |
| CTRL - PB+F | Central | 1.531E-06 | -2.1457625 | -2.8846703 | -1.406854691 |
| PB - F | Central | 2.467E-04 | -1.5833411 | -2.3222489 | -0.844433303 |
| PB - PB+F | Central | 6.046E-05 | -1.7525220 | -2.4914298 | -1.013614150 |
| F - PB+F | Central | 3.528E-01 | -0.1691808 | -0.9080887 | 0.569726961 |
| CTRL - PB | Inner half | 2.960E-02 | -0.8484301 | -1.5873379 | -0.109522333 |
| CTRL - F | Inner half | 3.239E-15 | -3.7629038 | -4.5018116 | -3.023995978 |
| CTRL - PB+F | Inner half | 2.434E-20 | -4.5595181 | -5.2984259 | -3.820610257 |
| PB - F | Inner half | 2.683E-10 | -2.9144736 | -3.6533815 | -2.175565838 |
| PB - PB+F | Inner half | 6.749E-15 | -3.7110879 | -4.4499957 | -2.972180116 |
| F - PB+F | Inner half | 3.817E-02 | -0.7966143 | -1.5355221 | -0.057706471 |
| CTRL - PB | Close to dentin | 1.625E-01 | -0.4412056 | -1.1801134 | 0.297702186 |
| CTRL - F | Close to dentin | 4.400E-05 | -1.7890580 | -2.5279658 | -1.050150146 |
| CTRL - PB+F | Close to dentin | 2.657E-07 | -2.3154096 | -3.0543174 | -1.576501824 |
| PB - F | Close to dentin | 1.451E-03 | -1.3478523 | -2.0867601 | -0.608944525 |
| PB - PB+F | Close to dentin | 2.058E-05 | -1.8742040 | -2.6131118 | -1.135296203 |
| F - PB+F | Close to dentin | 1.203E-01 | -0.5263517 | -1.2652595 | 0.212556130 |

**Table S4.** Mean calcium and carbon counts per second (cps) and Ca/C ratio in enamel determined by SEM–EDS.

| **Group** | **Mean Ca (cps)** | **Mean C (cps)** | **Ratio (Ca/C)** |
| --- | --- | --- | --- |
| **Control** | 12682 | 4825 | 2.63 |
| **Pb** | 12573 | 5967 | 2.11 |
| **F** | 11445 | 7139 | 1.60 |
| **Pb+F** | 11368 | 7286 | 1.56 |

| **Table S5.** Frequency of hypomineralized dentin near the enamel-dentin junction in mandibular third molars by experimental group (n=10 per group) detected by a calibrated examiner (Kappa of 0.95). | | |
| --- | --- | --- |
| **Group** | **Dentin Hypomineralization present** | **Dentin Hypomineralization absent** |
| **Control** | 0 | 10 |
| **Pb (30 ppm)** | 0 | 10 |
| **F (50 ppm)*** | 8 | 2 |
| **Pb + F*** | 10 | 0 |

*Differences between proportions: F vs Control or Pb groups: Cohen`s H of 2.21 (CI 95%: 1.34-3.09; Z-test: p-value of 7.37x10^-7^); Pb+F vs Control or Pb groups: Cohen`s H of 3.14 (CI 95%: 2.27-4.02; Z-test: p-value of 2.14x10^-12^). Other contrasts presented no statistically significant differences.

**References**

1 Bartlett JD, Beniash E, Lee DH, Smith CE (2004) Decreased mineral content in MMP-20 null mouse enamel is prominent during the maturation stage. J Dent Res 83:909–913. <https://doi.org/10.1177/154405910408301204>

2 De Sousa FB, Soares JD, Vianna SS (2013) Natural enamel caries: a comparative histological study on biochemical volumes. Caries Res 47:183–192. https://doi.org/10.1159/000345378

3 Perera T, Ranasinghe S, Alles N, Waduge R (2018) Effect of fluoride on major organs with different exposure times in rats. Environ Health Prev Med 23:17.

4 Weng Q, Yi F, Yu Y, Ge S, Liu S, Zhang Y (2021) Altered miRNA expression profiling in the enamel organ of fluoride-affected rat embryos. Ecotoxicol Environ Saf 210:111876. <https://doi.org/10.1016/j.ecoenv.2020.111876>

5 Peckham S, Awofeso N (2014) Water fluoridation: a critical review of the physiological effects of ingested fluoride as a public health intervention. Scientific World Journal 2014:293019. doi:10.1155/2014/293019

6 Adkins EA, Brunst KJ. 2021. Impacts of fluoride neurotoxicity and mitochondrial dysfunction on cognition and mental health: a literature review. Int J Environ Res Public Health. 18(21):11081. doi:10.3390/ijerph182111081

7 Brazil. Ministry of Health. Secretariat of Primary Health Care. Department of Community Health Strategies and Policies. General Coordination of Oral Health. Guide for recommendations on the use of fluorides in Brazil: updated 2024 version for public consultation. Brasília: Ministry of Health; 2024
